# Amplification of transglutaminase 2 enhances tumor-promoting inflammation in gastric cancers

**DOI:** 10.1038/s12276-020-0444-7

**Published:** 2020-05-28

**Authors:** Sung-Yup Cho, Yumi Oh, Eui Man Jeong, Sanghui Park, Dakeun Lee, Xiaorui Wang, Qiqi Zeng, Hongyu Qin, Fang Hu, Hui Gong, Xi Liu, Guanjun Zhang, Deukchae Na, Jieun Lee, Jeesoo Chae, Yun-Suhk Suh, Seong-Ho Kong, Hyuk-Joon Lee, Jong-Il Kim, Hansoo Park, Chengsheng Zhang, Han-Kwang Yang, Charles Lee

**Affiliations:** 1 Department of Biochemistry and Molecular Biology, Seoul National University College of Medicine, Seoul, Korea; 2 Department of Biomedical Sciences, Seoul National University College of Medicine, Seoul, Korea; 3 Cancer Research Institute, Seoul National University College of Medicine, Seoul, Korea; 4 Institute of Human-Environment Interface Biology, Seoul National University College of Medicine, Seoul, Korea; 5 Department of Pathology, Ewha Womans University College of Medicine, Seoul, Korea; 6 Department of Pathology, Ajou University School of Medicine, Suwon, Korea; 7 Precision Medicine Center, The First Affiliated Hospital of Xi’an Jiaotong University, Xi’an, China; 8 Department of Pathology, The First Affiliated Hospital of Xi’an Jiaotong University, Xi’an, China; 9 Ewha Institute of Convergence Medicine, Ewha Womans University Mokdong Hospital, Seoul, Korea; 10 Department of Life Science, Ewha Womans University, Seoul, Korea; 11 Department of Surgery, Seoul National University College of Medicine, Seoul, Korea; 12 Medical Research Center, Genomic Medicine Institute (GMI), Seoul National University, Seoul, Korea; 13 Department of Biomedical Science and Engineering, Gwangju Institute of Science and Technology (GIST), Gwangju, Korea; 14 The Jackson Laboratory for Genomic Medicine, Farmington, CT USA

**Keywords:** Tumour immunology, Cancer genomics, Gastric cancer

## Abstract

Tumor-promoting inflammation is a hallmark of cancer and is highly associated with tumor progression, angiogenesis, and metastasis. Tumor-associated macrophages (TAMs) are major drivers of tumor-promoting inflammation, but due to the complexity of the tumor microenvironment, the detailed regulatory mechanisms are still under investigation. Here, we investigated a novel role for transglutaminase 2 (TGM2) in the development of tumor-promoting inflammation and recruitment of TAMs to gastric cancer (GC) tissues. When estimated by array comparative genomic hybridization and droplet digital PCR, the copy numbers of the TGM2 gene were amplified in 13.6% (14/103) of GC patients and positively associated with TGM2 expression. Gene set enrichment analysis of expression microarray data for GC samples with high or low TGM2 expression showed that increased TGM2 expression was associated with tumor-promoting inflammation in GC. In addition, the expression of TGM2 was correlated with the expression of markers for macrophages, neutrophils, blood vessels, and lymphatic vessels. Overexpression of TGM2 in GC cells augmented the IL-1β-induced secretion of macrophage-recruiting chemokines and NF-κB activation. TGM2 protein levels were associated with the expression levels of the macrophage marker CD163 in human GC tissue samples. Moreover, GC patients with high expression of TGM2 had a worse prognosis than those with low expression of TGM2. These results suggest TGM2 as a novel regulator of the tumor microenvironment of GC and provide a promising target for constraining tumor-promoting inflammation.

## Stomach cancer: Enzyme promotes problematic inflammation

An enzyme linked to harmful inflammation that promotes tumor growth could be a target for stomach cancer treatment. The expression of the enzyme transglutaminase 2 (TGM2) has been shown to increase in several types of cancer, but its precise role has been unclear. Sung-Yup Cho at Seoul National University College of Medicine, Korea and co-workers measured copy numbers and expression levels of TGM2 in gastric cancer cell lines and in tissue samples from patients undergoing gastrectomy. They found that higher levels of TGM2 were closely associated with increases in various genes that promote the recruitment and activity of tumor-associated macrophages, malfunctioning white blood cells that drive tumor-promoting inflammation. In addition, stomach cancer patients with higher expression of TGM2 had poorer prognoses, indicating the potential therapeutic value of targeting this enzyme.

## Introduction

Unremitting inflammation is a hallmark of cancer and exhibits paradoxical effects on tumorigenesis and tumor progression^1,2^. There are two types of inflammation associated with cancer: (1) tumor-promoting inflammation, which promotes cell survival, aggressiveness and metastasis and is associated with chronic inflammation and T_H_2 responses; and (2) tumor-destroying inflammation, which induces cancer cell destruction and is related to acute inflammation and T_H_1 responses^1^. Tumor-promoting inflammation is characterized by a tumor microenvironment with increased levels of growth factors, angiogenic factors, and tissue-remodeling enzymes secreted from infiltrating immune cells^1^. Tumor-associated macrophages (TAMs) are essential drivers of tumor-promoting inflammation^3^. Increased infiltration of TAMs has been associated with an unfavorable prognosis in several types of cancers, including follicular lymphoma, Hodgkin lymphoma, breast cancer, and pancreatic cancer^3^.

Transglutaminase 2 (TGM2) is a calcium-dependent enzyme that mediates the posttranslational modification of proteins by incorporating lysine residues or polyamines into protein-bound glutamine residues^4^. The expression of TGM2 is increased in several types of cancers, including breast, ovarian, prostate, and pancreatic cancers^5^, and the enzymatic activity of TGM2 is augmented in various cancer-associated stress conditions, such as hypoxia, oxidative stress, endoplasmic reticulum stress, and chemotherapeutic treatment^6–9^. The expression of TGM2 is also increased in gastric cancer (GC) tissues, and TGM2 promotes cell proliferation, migration, and invasion in GC cell lines^10^. However, the roles of TGM2 in the regulation of the tumor microenvironment of GC have not been elucidated.

In this study, we demonstrated that the copy number of the TGM2 gene was amplified in a subset (13.6%) of GC samples and that increased TGM2 expression was associated with tumor-promoting inflammation mediated by recruiting macrophages to the tumor microenvironment. The association between TGM2 expression and tumor-promoting macrophages was verified by examining human GC tissue samples. These results identify novel roles for TGM2 in modulating the tumor microenvironment and tumor progression in GC.

## Materials and methods

### Gastric cancer patient sample collection and genomic DNA extraction

Frozen tissue samples of GC tissue and paired normal gastric tissue were obtained from individuals who underwent gastrectomy at Seoul National University Hospital. Total DNA was extracted from sections using the QIAamp DNA Mini Kit (QIAGEN). All samples were obtained with informed consent at the Seoul National University Hospital, and the study was approved by the institutional review board in accordance with the Declaration of Helsinki.

### Array comparative genomic hybridization (aCGH)

We designed a copy number variation (CNV)-targeted aCGH platform using the 1 M format on SurePrint G3 Human CGH Microarrays (Agilent Technologies). We conducted aCGH experiments and analyzed data according to the protocols described in a previous report^11^.

### Droplet digital PCR (ddPCR)

Extracted genomic DNA was restriction digested with the EcoRI enzyme (New England Biolabs) for 1 h at 37 °C. A 20-µL PCR mixture solution containing 1× ddPCR SuperMix (Bio-Rad), 1× probe and primer premix for the target gene and internal control gene, RNase P (final concentration of 250 nM for the probe and 900 nM for each primer; Applied Biosystems), and 10 ng of the restriction digested DNA was prepared. The reaction mixture and droplet generation oil (Bio-Rad) were loaded into a droplet generator (QX-200; Bio-Rad). The droplets were transferred to a 96-well PCR plate, and PCR was performed as follows: enzyme activation for 10 min at 95 °C; 40 cycles of 94 °C for 30 s, 60 °C for 1 min, and 98 °C for 10 min; enzyme deactivation for 10 min at 98 °C; and a hold at 4 °C (performed with a ramp rate of 2 °C/s in all steps). The PCR plate was placed in a droplet reader (Bio-Rad). After the reading, the copy number variation of each target gene was analyzed with Quanta software (Bio-Rad) on the droplet reader. The amplification threshold value was set at 2.5 for patient tissue specimens and 3.0 for cell lines.

### Cell culture

Gastric cancer cells were obtained from the Korean Cell Line Bank and maintained in RPMI 1640 medium (Life Technologies) containing 10% fetal bovine serum (Life Technologies). Penicillin (100 U/mL; Life Technologies) and streptomycin sulfate (100 μg/mL; Life Technologies) were added to all cell culture media. All cells were maintained in a humidified incubator with 5% CO_2_ at 37 °C.

### Quantitative real-time PCR

Total RNA was purified using the RNeasy Plus Mini Kit (QIAGEN) according to the manufacturer’s instructions. One microgram of total RNA was reverse transcribed into cDNA using Maxime RT PreMix (Intron Biotechnology) for 1 h at 45 °C. Quantitative real-time PCR was performed using SYBR Green PCR Master Mix (Applied Biosystems). Glyceraldehyde 3-phosphate dehydrogenase (GAPDH) was used as the internal control for normalization. The sequences of the primers for TGM2 were 5′-AGAAGAGCGAAGGGACGTACTG-3′ and 5′-AGTCTACCACGTCGGCATTGAC-3′, the sequences for the CCL2 primers were 5′-GTCTCTGCCGCCCTTCTGT-3′, and 5′-TTGCATCTGGCTGAGCGAG-3′, the sequences for the CXCL10 primers were 5′-GTGGCATTCAAGGAGTACCTC-3′ and 5′-GCCTTCGATTCTGGATTCAG-3′, and the sequences for the GAPDH primers were 5′-CGCTCTCTGCTCCTCCTGTT-3′ and 5′-CCATGGTGTCTGAGCGATGT-3′.

### Western blot analysis

Cells were lysed in RIPA buffer (Thermo Scientific) containing a protease inhibitor cocktail (Roche) and phosphatase inhibitor cocktail (Roche) and were centrifuged at 20,000 × *g* for 10 min at 4 °C. After determination of the protein concentration in the cell extract by the BCA method (Thermo Scientific), 20 μg of protein was resolved by SDS-PAGE and transferred to a polyvinyl difluoride membrane. Membranes were blocked for 1 h with 5% skim milk in Tris-buffered saline and then incubated with anti-TGM2^12^, anti-phospho-NF-κB (Ser276 and Ser536, Cell Signaling Technology), anti-NF-κB (Cell Signaling Technology), and anti-Actin (Sigma-Aldrich Corporation) antibodies. The membranes were washed and incubated with a horseradish peroxidase-conjugated secondary antibody, followed by enhanced chemiluminescence development according to the manufacturer’s instructions (Pierce).

### Microarray data analysis and gene set enrichment analysis (GSEA)

Microarray data sets of a Helicobacter-induced gastric cancer mouse model (GSE13873) and gastric cancer tissue samples (GSE27342) were downloaded from GEO (www.ncbi.nlm.nih.gov/geo/). Each Affymetrix data set was background adjusted and normalized with the Robust Multichip Averaging (RMA) algorithm in the Affy package using R ver. 3.1.1^13^. GSEA was performed using the javaGSEA desktop application (GSEA v2.1.0)^14,15^. The gene sets from gene ontology (GO) biological process and motif gene sets for transcription factor targets were used, and the gene sets with fewer than 15 genes or more than 500 genes were excluded. *P*-values were calculated by permuting the data 1000 times to find enriched gene sets. The GSEA software produces an enrichment score (ES), a normalized ES (NES), a nominal *P*-value and a false discovery rate (FDR; *Q*-value). Gene sets that were upregulated or downregulated with a *Q*-value < 0.05 were considered significant.

### Analysis of a data set from The Cancer Genome Atlas (TCGA)

To investigate the genomic and transcriptomic characteristics of another GC cohort, we downloaded data from TCGA (Stomach adenocarcinoma (TCGA, Firehose Legacy), *n* = 478)^16,17^. We obtained mutation, copy number alteration, and gene expression data using the cBioPortal database (http://www.cbioportal.org).

### Estimation of infiltrating immune cells using CIBERSORT analysis

To investigate the fractions of infiltrating immune cells in GC tissues, cell-type identification by estimating relative subsets of known RNA transcripts (CIBERSORT) (https://cibersort.stanford.edu/) analysis was conducted^18^. CIBERSORT is an analytical tool that provides an estimation of the abundances of member cell types in a mixed cell population using gene expression data^18^. The proportion of each infiltrating immune cell was calculated based on the absolute mode.

### In vitro THP-1 cell migration assay

An in vitro THP-1 cell migration assay was performed using a CytoSelect™ 96-well cell migration assay kit (5-µm pore, Cell Biolabs, Inc.) according to the manufacturer’s instructions. Briefly, conditioned medium was prepared from MKN-45 cells, which were transfected with control or TGM2-expressing plasmids and treated with 10 ng/mL IL-1β for 24 h after overnight serum starvation. Medium aliquots (150 µL) were added to the wells of the feeder tray. THP-1 cells, which were incubated in serum-free medium for 18 h, were seeded in the upper well of the chamber (4 × 10^5^ cells in 100 µL RPMI-1640 medium) and then incubated for 2 h. The medium in the feeder tray was utilized to determine the amount of migrated THP-1 cells. Migratory THP-1 cells dissociated from the membrane were lysed and quantified using the CyQuant® GR fluorescent dye.

### Reporter assay

NF-κB activity was monitored using 6× NFκB reporter constructs. Cells were transfected with 6× NFκB promoter-firefly luciferase reporter constructs and then treated with 10 ng/mL IL-1β for 24 h after overnight serum starvation. The cells were harvested and assayed for luciferase activity using the Dual-Luciferase Reporter Assay System (Promega). The cells were cotransfected with the pRL-TK Renilla luciferase vector as the internal control. The firefly luciferase activity was normalized to the Renilla luciferase activity.

### Immunohistochemistry (IHC)

The tissue specimens used for IHC were obtained from patients diagnosed with gastric cancer at the First Affiliated Hospital of Xi'an Jiaotong University. A total of 59 formalin-fixed, paraffin-embedded (FFPE) gastric cancer tissue specimens (30 intestinal-type and 29 diffuse-type specimens based on the Lauren classification system) were deidentified and approved by the IRB for use in this study. Immunohistochemical staining was performed on 4-µm FFPE slides prepared by the Department of Pathology at the First Affiliated Hospital of Xi'an JiaoTong University. In brief, the slides were deparaffinized with xylene and dehydrated with ethanol. The primary antibodies used in this study included anti-TGM2 (MA5–12739, 1:150 dilution, Invitrogen) and anti-CD163 (MA5–11458, 1:30 dilution, Invitrogen). Phosphate-buffered saline was used in place of the primary antibody as a negative control. Detection was carried out using the HRP (Mouse/Rabbit) IHC Kit (Maixin Biotech, KIT-9922, China) and DAB Plus Kit (Maixin Biotech, DAB-2032, China). The slides were scanned with a microscopy imaging system (Leica SCN400). Endothelial cells and reactive histiocytes were used as positive controls for TGM2 and CD163 staining, respectively.

TGM2 immunoreactivity was divided into three categories according to the staining intensity: (1) negative or weak expression (−, −/+, and +); (2) moderate expression (++ and +++); and (3) strong expression (++++ and +++++). The assessment of CD163 immunoreactivity was performed for macrophages in cancer tissues and divided into the following two groups: low expression (−, −/+, and +) and high expression (++, +++, ++++, and +++++).

### Statistical analysis

Statistical calculations were performed using Prism 4.0 (GraphPad). Differences between two variables and multiple variables were assessed by Student’s *t*-test and one-way ANOVA with Tukey's multiple comparison test, respectively. Associations between two discrete variables were estimated by Fisher's exact test. Correlations between two continuous variables were estimated by linear regression analysis. Differences in overall survival between two groups were analyzed by the log-rank test. A difference was considered significant if the *P*-value was less than 0.05.

## Results

### Copy number amplification of TGM2 in gastric cancer patients

**Fig. 1:**
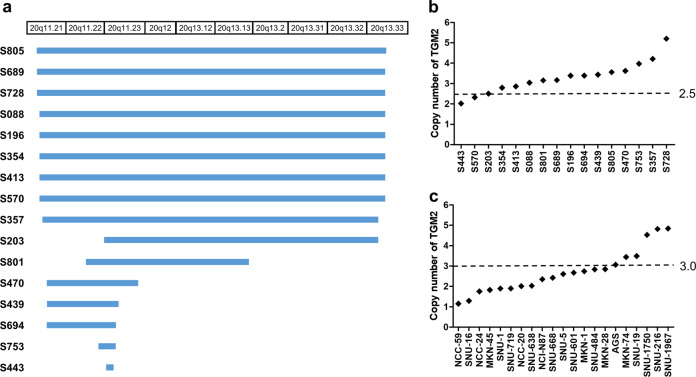
Copy number amplification of TGM2 in gastric cancer (GC). **a** The chromosomal locations of TGM2-containing amplicons in 16 TGM2-amplified GC patients estimated by array comparative genomic hybridization (aCGH). **b**, **c** Estimated copy numbers of TGM2 in 16 TGM2-amplified GC patients (**b**) and 21 GC cell lines (**c**) evaluated by droplet digital PCR (ddPCR). Error bars indicate the Poisson 95% confidence interval for each determination. The dashed line indicates the ddPCR threshold cut-off of 2.5 or 3.0 copies for calling a sample TGM2 amplified in the GC patients (**b**) and GC cell lines (**c**), respectively.

In a previous study, we performed aCGH of DNA from 103 Korean GC patients to assess copy number alterations (CNAs) and reported copy number amplifications of several genes, including *MYC*, *ERBB2*, *BCL2L1*, and *GRB7*^11^. In this aCGH data set, we found copy number amplification of *TGM2* in 15.5% (16/103) of the 103 GC patients (Fig. 1a, Table S1). In other genes in the transglutaminase family, copy number amplifications were detected at a very low rate (Table S1). There were no significant differences between *TGM2*-amplified and nonamplified patients with respect to clinical characteristics such as age, sex, tumor stage, and tumor location (Table S2). TGM2 amplification was detected in 22% (11/50) and 12.5% (4/32) of patients with the intestinal and diffuse type, respectively, but the difference in terms of Lauren classification was not statistically significant (*P* = 0.6362) (Table S2). Using ddPCR, we validated the observed amplification of the *TGM2* gene in 14 of the 16 patients detected by aCGH (more than or equal to 2.5 copies considering tumor cellularity; Fig. 1b). In addition, targeted ddPCR showed amplification of the *TGM2* gene in 23.8% (5/21) of available GC cell lines (more than or equal to three copies; Fig. 1c).

**Fig. 2:**
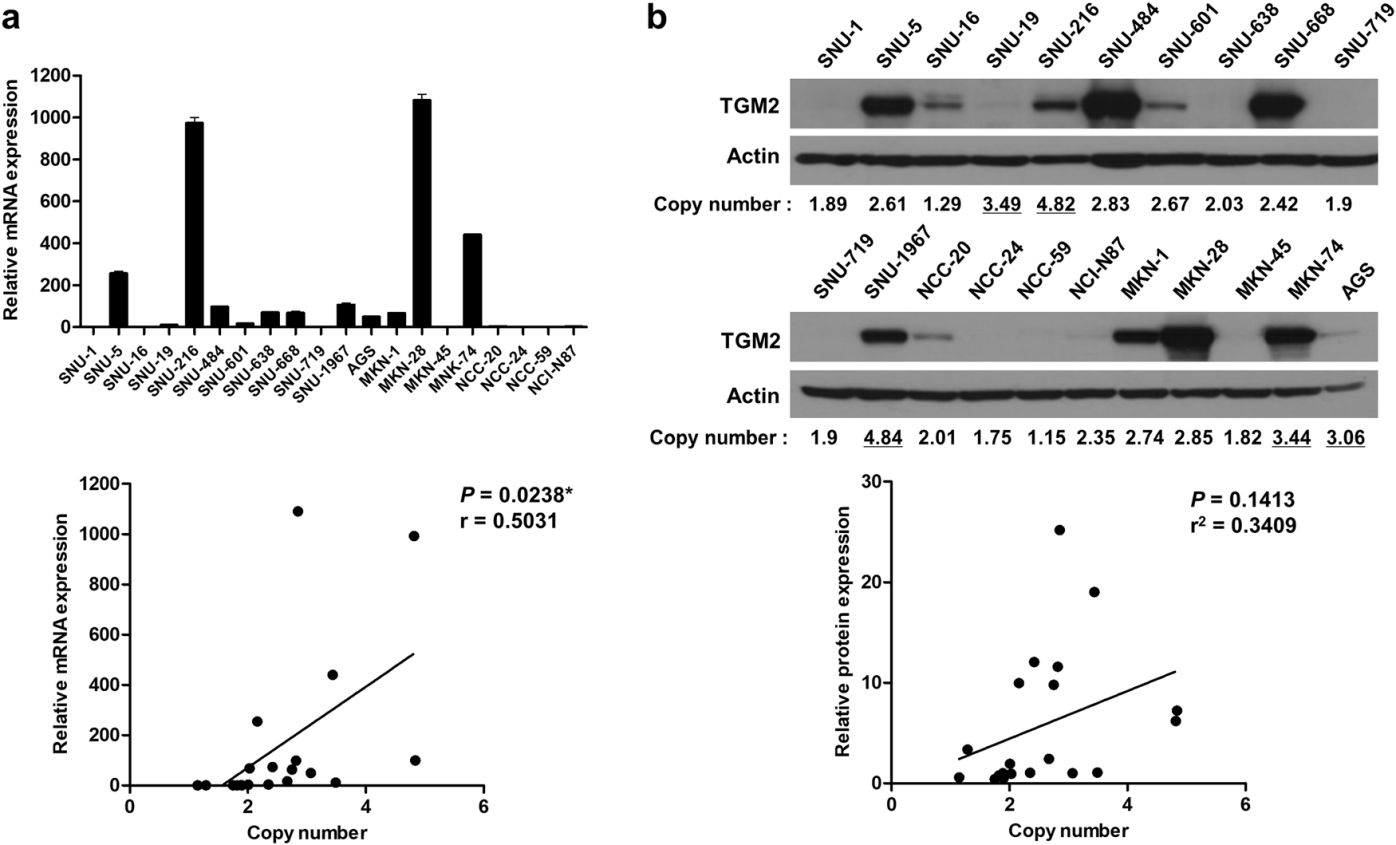
The correlation between TGM2 expression levels and TGM2 copy numbers in GC cell lines. **a** The mRNA expression levels and copy numbers of TGM2 in 20 GC cell lines. The messenger RNA expression levels of TGM2 were quantified by real-time PCR (upper panel), and the copy number values of TGM2 were estimated by ddPCR. The lower panel shows the correlation between the mRNA expression levels and copy numbers of TGM2 in the GC cell lines, and the *P*-value determined by linear regression (*P < 0.05) and Pearson correlation coefficient (*r*) are indicated. **b** Protein expression levels and copy numbers of TGM2 in 20 GC cell lines. The protein levels of TGM2 were evaluated by western blot analysis, and the copy number values of TGM2 were estimated by ddPCR (upper panel). The underlined numbers represent the TGM2-amplified samples (threshold cut-off of 3.0 copies). The lower panel shows the correlation between the protein expression levels and copy numbers of TGM2 in the GC cell lines. The protein expression levels were quantified by densitometric analysis for western blotting using ImageJ. The correlation between gene expression and copy number was estimated by linear regression analysis, and the Pearson correlation coefficient (*r*) is indicated.

In GC cell lines, the mRNA expression levels of TGM2 correlated well with the copy number of the *TGM2* gene determined by ddPCR (*P* = 0.0238; Fig. 2a). The expressed amount of the TGM2 protein in GC cells showed a positive correlation with the copy number of the *TGM2* gene determined by ddPCR (Pearson correlation coefficient = 0.34; Fig. 2b). We also investigated another GC cohort from TCGA (Stomach adenocarcinoma (TCGA, Firehose Legacy), *n* = 478; http://www.cbioportal.org)^16,17^ and found that the samples from patients with copy number gain and amplification of the TGM2 gene determined by the GISTIC algorithm exhibited higher mRNA expression levels of TGM2 than those from patients with a diploid TGM2 gene (Fig. S1a, b). These results suggest that copy number amplification of the TGM2 gene is associated with increased expression of TGM2.

### TGM2 is associated with tumor-promoting inflammation in gastric cancers

To investigate the association between TGM2 expression and gastric cancers, we examined the mRNA expression of transglutaminase family genes in mouse gastric cancer models and human gastric cancer tissue specimens using microarray data sets from the Gene Expression Omnibus (GEO) database (https://www.ncbi.nlm.nih.gov/geo). In *Helicobacter felis*-infected model mice (GSE13873)^19^, which develop premalignant lesions such as gastric atrophy, compensatory epithelial hyperplasia and intestinal metaplasia, the expression of TGM2 was significantly increased in hyperplastic and metaplastic lesions compared with uninfected control tissue (Fig. S2). However, the expression of other transglutaminases was not increased in premalignant lesions (Fig. S2). In addition, analyses of gene expression in 80 paired GC/reference normal tissue specimens (GSE27342) showed that the expression of TGM2 was significantly increased in the GC tissue samples compared to the matched normal tissue samples (Fig. S3). In contrast, other transglutaminases including TGM1, TGM4, TGM5, TGM6, and TGM7 exhibited a significant decrease in gene expression in the gastric cancer tissue samples (Fig. S3). These results demonstrated that increased gene expression of TGM2 was highly associated with the development of GC.

**Fig. 3:**
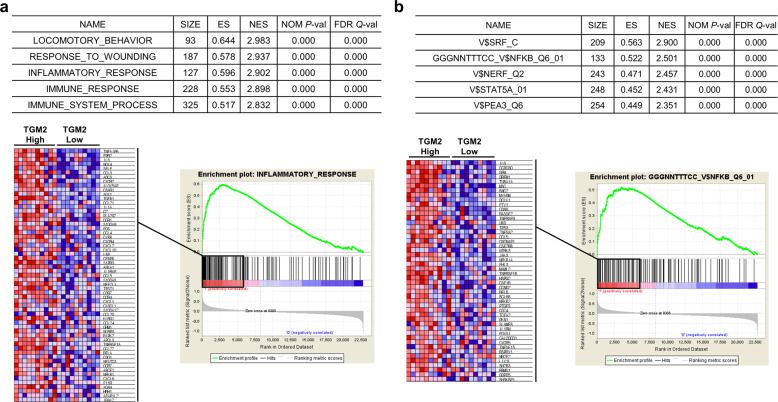
Gene set enrichment analysis (GSEA) of the mRNA profiles for GC samples with the highest expression levels of TGM2 (TGM2_H) and lowest expression levels of TGM2 (TGM2_L). **a** GSEA using gene ontology (GO) gene sets to compare the TGM2_H (*n* = 10) and TGM2_L (*n* = 10) groups. Microarray data were downloaded from Gene Expression Omnibus (GEO; GSE27342). The upper table shows the list of the top 5 enriched gene sets in the TGM2_H group with the enrichment score (ES), normalized enrichment score (NES), nominal *P*-value (NOM *P*-val) and false discovery rate (FDR *Q*-val). The lower panel represents the GSEA score curve and enriched genes for the “INFLAMMATORY_RESPONSE” gene set. Phenotype “1” represents TGM2_H samples, and phenotype “0” represents TGM2_L samples. **b** GSEA using gene sets from transcription factor-binding motif to compare the TGM2_H (*n* = 10) and TGM2_L (*n* = 10) groups. Microarray data were downloaded from Gene Expression Omnibus (GEO; GSE27342). The upper table shows the list of the top 5 enriched gene sets in the TGM2_H group with the enrichment score (ES), normalized enrichment score (NES), nominal *P*-value (NOM *P*-val) and false discovery rate (FDR *Q*-val). The lower panel represents the GSEA score curve and enriched genes for the gene set with NF-κB-binding motifs in the promoter region. Phenotype “1” represents TGM2_H samples, and phenotype “0” represents TGM2_L samples.

To identify the roles of TGM2 in GC carcinogenesis, we investigated the enriched gene sets in GC samples with high expression of TGM2. Using the previously analyzed microarray data for 80 gastric cancer samples from the GEO database (GSE27342), we selected the ten samples with the highest expression levels of TGM2 (TGM2_H) and ten samples with the lowest expression levels of TGM2 (TGM2_L) and performed GSEA^15^ comparing these two groups (Fig. S4). When gene sets from gene ontology (GO) biological processes were applied, gene sets associated with inflammation, such as INFLAMMATORY_RESPONSE, IMMUNE_RESPONSE, and IMMUNE_SYSTEM_PROCESS, were highly enriched in the TGM2_H group (Fig. 3a). Analysis using motif gene sets for transcription factor targets showed that the gene set with NF-κB-binding motifs in the promoter region was enriched in the TGM2_H group (Fig. 3b). NF-κB is a transcription factor that is considered one of the master regulators of inflammation^20^. In addition, 327 genes that showed correlated expression with TGM2 (Pearson correlation coefficient ≥ 0.3) in the GC cohort from the TCGA (Stomach adenocarcinoma (TCGA, Firehose Legacy, *n* = 478)) (Table S3) were highly enriched in gene sets associated with inflammation, including IMMUNE_RESPONSE, INFLAMMATORY_RESPONSE and INNATE_IMMUNE_RESPONSE (Table S4), when analyzed with the DAVID bioinformatic resources (http://david.abcc.ncifcrf.gov)^21^. These results suggest that TGM2 expression is highly associated with inflammation in GC.

**Fig. 4:**
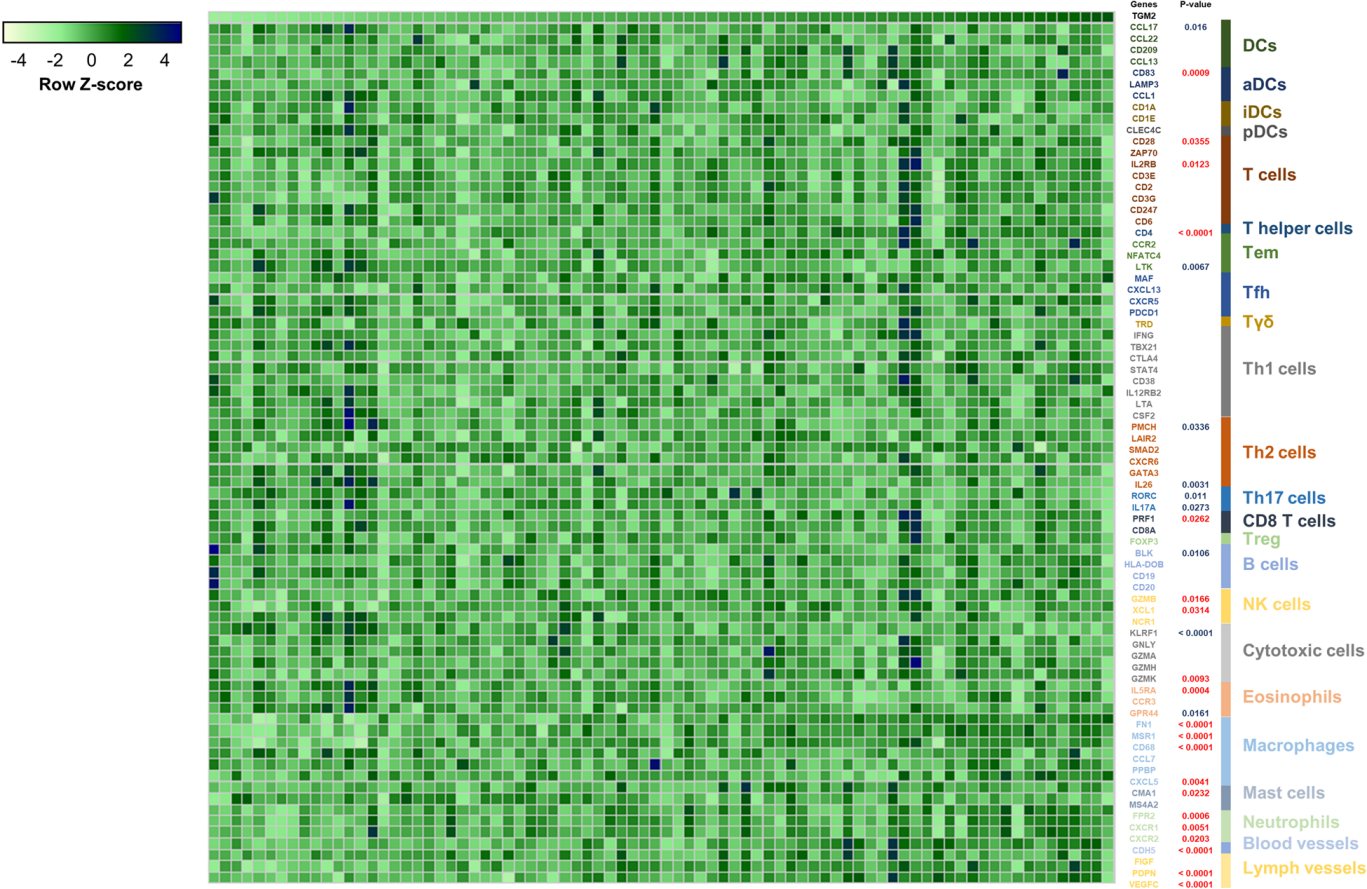
Correlations between TGM2 expression and immune cell type-specific gene expression in 80 gastric cancer tissue specimens. Microarray data were downloaded from Gene Expression Omnibus (GEO; GSE27342), and the heatmap represents the matrix of gene expression of TGM2 and 76 immune cell type-specific genes representing 23 cell types, which are shown in colored gene names and bars (right). Each column represents an individual GC sample, and each row denotes a gene. The correlation between TGM2 expression and immune cell type-specific gene expression was estimated by linear regression analysis, and the *P*-values for genes that were significantly correlated with TGM2 expression are depicted (*P* < 0.05; red: positive correlation, blue: negative correlation). DCs dendritic cells, aDCs activated DCs, iDCs immature DCs, pDCs plasmacytoid DCs, Tem effector memory T cell, Tfh follicular helper T cell, Tγδ gamma delta T cell, and Treg regulatory T cell.

Next, we investigated the role of TGM2 in GC inflammation by analyzing immune cell signatures associated with TGM2 expression. When the marker gene expression of immune cell types published in a previous report^22^ was analyzed, TGM2 expression was highly correlated with the expression of markers for macrophages, neutrophils, blood vessels, and lymphatic vessels (Figs. 4, S5), and these compartments were highly associated with tumor-promoting inflammation^1^. A detailed marker study revealed that TGM2 expression was highly correlated with the expression of M1 and M2 macrophage markers, N1 and N2 neutrophil markers, and markers for macrophage and neutrophil recruitment (Fig. S6). Estimating the abundances of 22 immune cell types from expression microarray data using the CIBERSORT algorithm^18^ also showed that the infiltration of M1 macrophages, M2 macrophages and neutrophils was positively correlated with the expression of TGM2 in GC tissue samples (Fig. S7a, b) and that the macrophage M1/M2 ratio was positively correlated with TGM2 expression (Pearson correlation coefficient = 0.1819; Fig. S7c). In the GC sample cohort from the TCGA (Stomach adenocarcinoma (TCGA, Firehose Legacy, *n* = 478)), the levels of several markers for macrophages (FN1, MRS1, CD68, and CCL7), blood vessels (CDH5) and lymphatic vessels (VEGFC) exhibited positive correlations with TGM2 expression (Fig. S8). Taken together, these data demonstrated that TGM2 expression was highly connected with tumor-promoting inflammation.

### TGM2 promotes macrophage recruitment to gastric cancer tissues

**Fig. 5:**
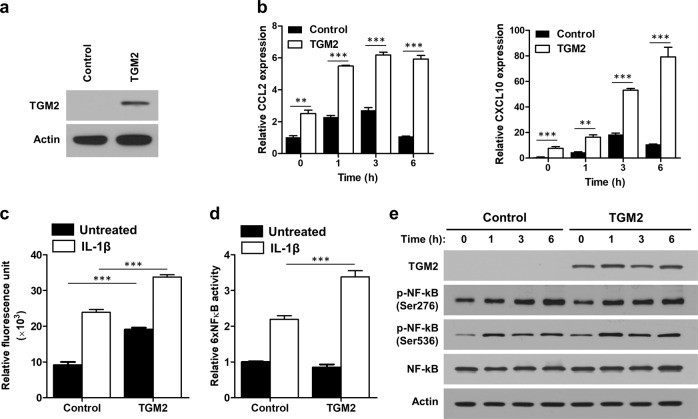
Regulation of interleukin-1β (IL-1β)-induced chemokine expression in gastric cancer cells by TGM2. **a** Overexpression of TGM2 in MKN-45 cells. The protein expression levels of TGM2 were evaluated by western blotting. **b** mRNA levels of CCL2 and CXCL10 in IL-1β (10 ng/mL)-treated MKN-45 cells. The mRNA expression of CCL2 and CXCL10 in TGM2-overexpressing and control cells was measured by real-time PCR at the indicated times. Asterisks indicate statistically significant differences (**P < 0.01; ***P < 0.001) compared to the control cells. **c** THP-1 macrophage migration induced by conditioned medium from MKN-45 cells. TGM2-overexpressing and control MKN-45 cells were treated with IL-1β (10 ng/mL), and the chemotactic effect of conditioned medium on THP-1 cells was estimated with a cell migration assay. Migrated cells were lysed and quantified using a fluorescent dye. Asterisks indicate statistically significant differences (***P < 0.001) compared to control cells. **d** The activity of an NF-κB-responsive element (6 × NFκB) after IL-1β treatment (10 ng/mL). The activity of the NF-κB-responsive element was estimated using a luciferase reporter assay to assess TGM2-overexpressing and control MKN-45 cells. Asterisks indicate statistically significant differences (***P < 0.001) compared to control cells. **e** Time-dependent changes in the protein levels of NF-κB after IL-1β treatment (10 ng/mL). The protein levels of total and phospho-NF-κB (Ser276 and Ser536) in TGM2-overexpressing and control MKN-45 cells were evaluated by western blotting at the indicated times.

Because macrophages in tumors are the major source of inflammatory mediators involved in tumor-promoting inflammation^23^, we investigated the roles of TGM2 in macrophage recruitment by tumor cells. Overexpression of TGM2 increased the basal expression levels of macrophage-recruiting chemokines such as C–C motif chemokine ligand 2 (CCL2) and C–X–C motif chemokine ligand 10 (CXCL10)^24^, and induction of these chemokines by interleukin-1β (IL-1β) treatment was substantially enhanced in TGM2-overexpressing MKN-45 cells compared with control cells (Fig. 5a, b). In addition, the IL-1β-mediated induction of CCL2 was significantly increased in GC cell lines with high TGM2 expression compared to GC cell lines with low TGM2 expression (Fig. S9). Conditioned medium from TGM2-overexpressing MKN-45 cells showed a stronger chemotactic effect on THP-1 macrophages than that from control cells (Fig. 5c). Since NF-κB is activated by proinflammatory cytokines, including IL-1β, in cancer cells^25^, we investigated the effect of TGM2 expression on NF-κB activity after IL-1β treatment. TGM2 overexpression augmented NF-κB activation induced by IL-1β treatment (Fig. 5d). However, the basal and IL-1β-induced phosphorylation levels of NF-κB at Ser276 and Ser536, which are associated with NF-κB activation^26^, were not altered by TGM2 overexpression (Fig. 5e), suggesting that the enhanced activity of NF-κB seen with TGM2 overexpression is not associated with the phosphorylation status of NF-κB.

### High expression of TGM2 is associated with macrophage infiltration and a poor prognosis in GC patients

**Fig. 6:**
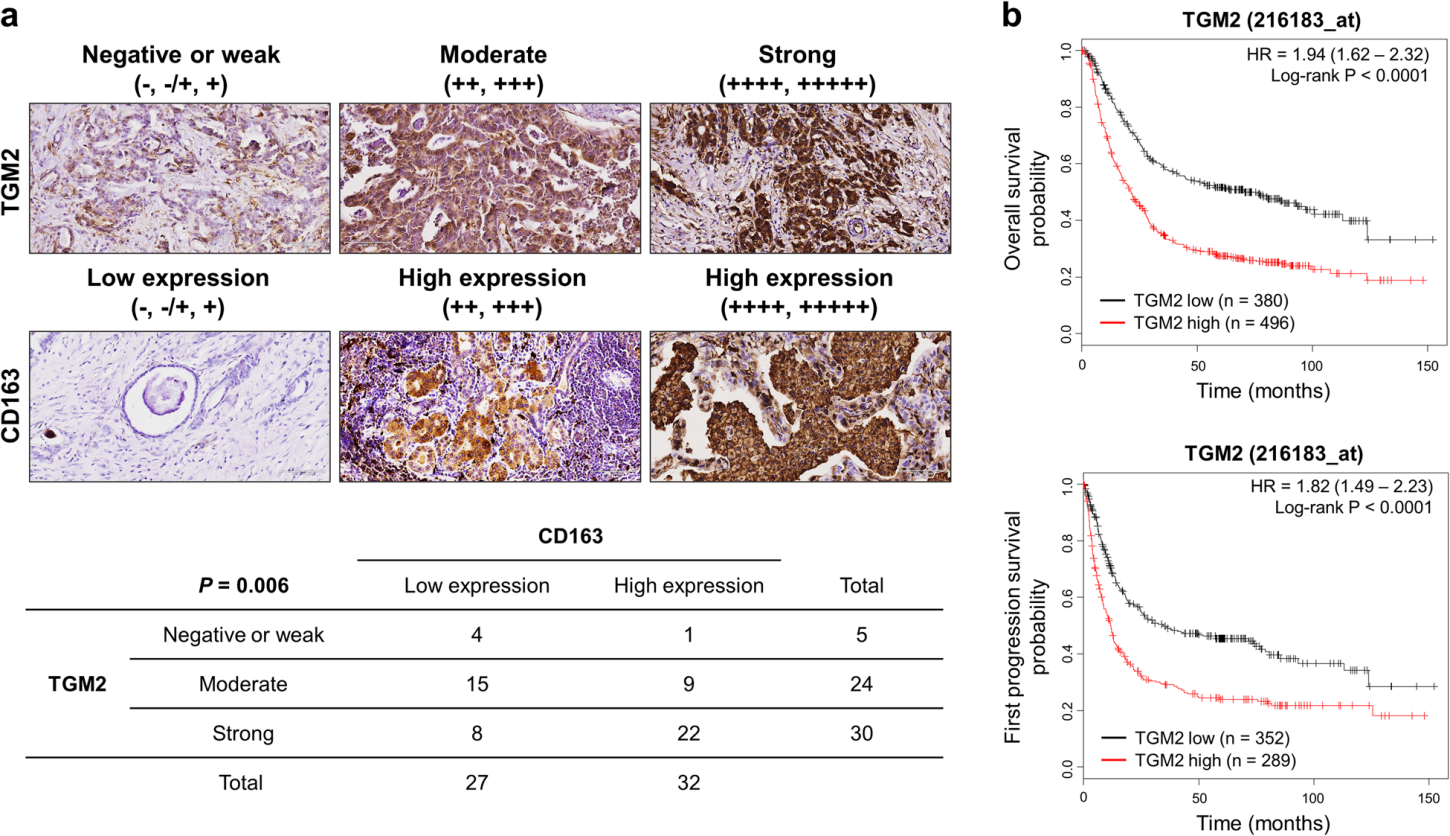
Association between TGM2 expression and macrophage infiltration in gastric cancer (GC) tissue samples. **a** Immunohistological analysis of TGM2 and the macrophage marker CD163 expression in GC tissue specimens. The upper panel shows representative microscopy images for immunohistological analysis of samples classified by the immunoreactivity of TGM2 and CD163. The lower table summarizes the expression levels of TGM2 and CD163 in 59 GC tissue samples. **b** Effect of TGM2 mRNA expression on the overall and first progression survival of GC patients. Kaplan–Meier plots were calculated for the overall (upper panel) and first progression survival (lower panel) of GC patients with high TGM2 expression (red line) or low TGM2 expression (black line) using the online bioinformatic tool Kaplan–Meier Plotter (https://kmplot.com/analysis/).

Next, the association between the protein expression of TGM2 and intratumoral infiltration of macrophages was investigated in human GC tissue specimens. In cancer tissue samples with negative/weak or moderate expression of TGM2 in GC cells, 20.0% (1/5) and 37.5% (9/24) of the samples, respectively, showed high expression of the macrophage marker CD163 (Fig. 6a). However, in cancer tissue samples with strong expression of TGM2 in GC cells, 73.3% (22/30) of the samples showed high expression of the macrophage marker CD163 (Fisher's exact test, *P* = 0.006; Fig. 6b). These results suggest that strong expression of TGM2 is associated with increased macrophage infiltration into gastric tumor tissues.

Finally, we performed survival analysis of GC patients with respect to the expression level of TGM2 using the online bioinformatic tool Kaplan–Meier Plotter (https://kmplot.com/analysis/)^27^. When the patients were divided into two groups based on their TGM2 expression, the group with high TGM2 expression had a significantly worse prognosis than the group with low TGM2 expression for both overall and first progression survival (*P* < 0.0001; Figs. 6b, S10a). In addition, the GC cohort from the TCGA also showed a poor prognosis for the high TGM2 expression group compared to the low TGM2 expression group when analyzed by Kaplan–Meier Plotter (*P* = 0.017 and *P* = 0.029 for overall and relapse-free survival, respectively; Fig. S10b). These data suggest that high expression of TGM2 is associated with a poor prognosis, probably due to enhanced tumor-promoting inflammation.

## Discussion

Inflammation has been suggested as an underlying enabling characteristic of cancer as it fosters cancer hallmark functions such as sustained proliferation, resistance to cell death, angiogenesis, and metastasis^2^. The tumor-promoting effect of inflammation is mainly elicited by factors secreted from tumor-infiltrating immune cells, and TAMs are an essential component in this process^3^. Our study demonstrated that copy number amplification of TGM2 in GC was highly associated with the recruitment of macrophages into the tumor microenvironment, leading to enhanced tumor-promoting inflammation.

TAMs originate from either embryonic precursors with a peripheral localization or circulating monocytes^28,29^. Macrophages have principally been classified into two activation statuses: the M1 type, which is induced during the type 1 immune response and associated with bacteria-killing and tissue-damaging properties, and the M2 type, which develops during the type 2 immune response and is related to parasite-resistant and tissue-remodeling properties^3^. Although there is intertumoral and intratumoral diversity among TAMs, polarization toward the M2 type and immunosuppressive features, which are usually estimated by the expression of the hemoglobin scavenger receptor CD163, seem to be shared characteristics of most cancers^3^. TAMs participate in tumor progression by secreting growth factors that stimulate the proliferation of cancer cells, producing extracellular matrix-digesting enzymes that increase tumor cell dissemination, generating reactive oxygen species that induce genomic instability in cancer cells, and providing angiogenic factors that promote angiogenesis and lymphangiogenesis^3^. Therefore, targeting the function of TAMs can be a relevant strategy for cancer therapies.

In many cancer types, increased infiltration of TAMs is associated with a poor prognosis^30,31^. A high degree of TAM infiltration is associated with an advanced tumor grade and unfavorable outcome in breast cancers^32^, bladder cancers^33^, pancreatic cancers^34^, and lymphomas^35,36^. In GC patients, TAM numbers in tumor tissue are highly correlated with serosa invasion, metastasis, angiogenesis and lymphangiogenesis^37^, and a meta-analysis of 5 studies including 447 patients showed that a high density of TAMs was significantly correlated with poor overall survival (risk ratio = 1.54 (95% CI 1.24–2.16))^31^. Therefore, the TAM density of the tumor microenvironment can be regarded as a prognostic factor in GC patients.

Overexpression of TGM2 has been reported in many cancer types, and high expression levels of TGM2 are highly correlated with metastasis, drug resistance and a poor survival rate in cancer patients^5^. Moreover, TGM2 has been suggested to be involved in epithelial–mesenchymal transition (EMT) and the cancer stem cell phenotype, which are highly associated with aggressive cancer phenotypes, in breast, ovarian and squamous cell carcinomas^38–40^. TGM2 is also involved in the PI3K/Akt survival pathway by activating FAK or c-Src^41,42^, the TGF-β signaling pathway by crosslinking latent TGF-β^43^, and NF-κB signaling pathway activation^44^. In GC, TGM2 was reported to promote the proliferation, migration and invasion of cancer cells by activating the ERK 1/2 pathway^10^. These data indicate that the expression of TGM2 is associated with an aggressive phenotype in cancer cells. Our study provides additional mechanisms involving TGM2 in cancer aggressiveness through enhancing tumor-promoting inflammation.

TGM2 also plays roles in the development of inflammatory diseases, such as celiac disease, inflammatory bowel disease, osteoarthritis, and idiopathic inflammatory myopathies^45^. One of the major molecular mechanisms underlying the involvement of TGM2 in inflammation is the activation of the NF-κB signaling pathway^45^. However, the exact molecular mechanism by which TGM2 activates NF-κB is somewhat controversial. TGM2 was reported to activate NF-κB by crosslinking and inhibiting IκBα, resulting in the nuclear translocation and activation of NF-κB^46^. Other studies suggested that TGM2 activated NF-κB via a noncanonical pathway independent of IκB kinase^47,48^. Our data showed that TGM2 overexpression enhanced the activity of NF-κB in IL-1β-treated GC cells, suggesting an activating role for TGM2 in the NF-κB pathway. However, the activity-related phosphorylation levels of NF-κB were not affected by TGM2 overexpression. Therefore, the detailed molecular mechanism by which TGM2 mediates NF-κB activation needs to be investigated in further studies.

In addition to cancer cells, macrophages express TGM2, and TGM2 reportedly participates in macrophage phagocytosis of apoptotic cells^49,50^. Macrophages from TGM2-knockout mice show a defect in the clearance of apoptotic cells created by failing to perform integrin β_3_-mediated formation of engulfing portals, resulting in the development of age-dependent autoimmunity^49,50^. In addition, TGM2 is suggested to be the marker of Th2-IL-4-activated macrophages (M2), which are implicated in the modulation of inflammation and repair^51^. Given that TGM2 expressed in macrophages also plays a modulatory role in tissue inflammation, targeting TGM2 in GC could be an effective strategy for modulating tumor-promoting inflammation by regulating both cancer cells and macrophages.

## Supplementary information

Supplementary information
