# Supplementary material for: Amplification of transglutaminase 2 enhances tumor-promoting inflammation in gastric cancers
Source: Exp Mol Med. 2020 May 28;52(5):854–64. doi: 10.1038/s12276-020-0444-7 (PMC7272405; doi:10.1038/s12276-020-0444-7)
Supplement: Supplementary file 1 — Supplementary information [file 12276_2020_444_MOESM1_ESM.pdf]

Supplementary Figures

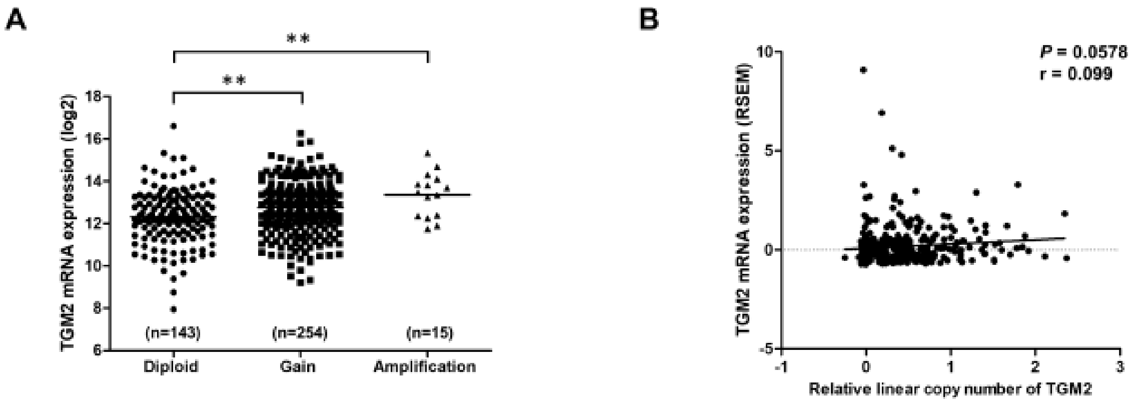

Supplementary Figure S1. The mRNA expression levels of TGM2 according to copy number alterations of TGM2 gene in The Cancer Genome Atlas (TCGA) gastric cancer cohort. (A) The TGM2 mRNA expression levels in gastric cancer patients with diploid, gain, and amplification of TGM2 gene. Data were downloaded from GC cohort dataset from TCGA database (Stomach adenocarcinoma (TCGA, Firehose Legacy), n = 478; http://www.cbioportal.org). Copy numbers of TGM2 were determined using GISTIC 2.0 algorithm and mRNA expression of TGM2 were estimated by RNA Seq V2 RSEM algorithm. Asterisks indicate statistically significant differences (**, $P < 0.01$) compared to TGM2 diploid group. (B) Linear correlation between TGM2 copy number and TGM2 mRNA expression in TCGA dataset (Stomach adenocarcinoma (TCGA, Firehose Legacy)). P-value by linear regression and Pearson correlation coefficient (r) were indicated.

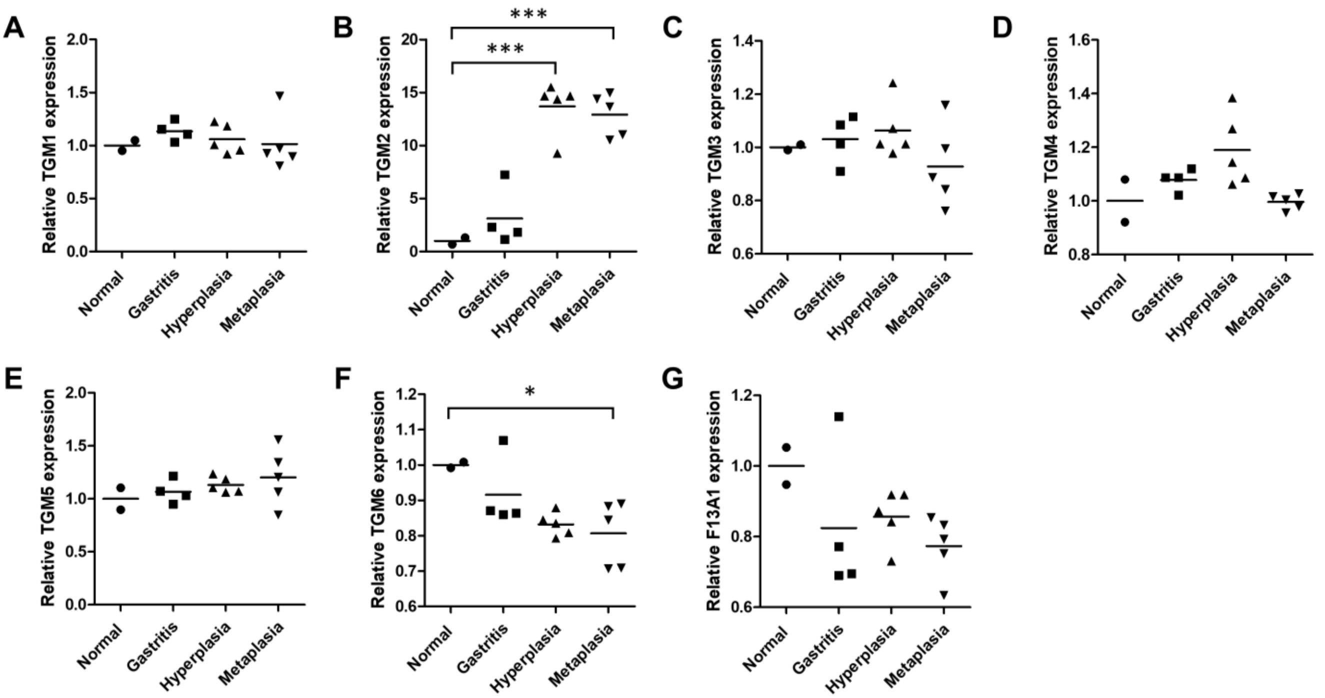

Supplementary Figure S2. The mRNA expression levels of transglutaminase family in Helicobacter-induced gastric cancer mouse model. (A-G) The mRNA expression levels of TGM1 (A), TGM2 (B), TGM3 (C), TGM4 (D), TGM5 (E), TGM6 (F), and F13A1 (G) in *Helicobacter felis*-infected mice models. Gene expression microarray data were downloaded from Gene Expression Omnibus (GEO) database (https://www.ncbi.nlm.nih.gov/geo; GSE13873). Relative gene expression levels were estimated compared to expression levels of normal gastric tissues, and asterisks indicate statistically significant differences (*, $P < 0.05$; ***, $P < 0.001$) compared to normal gastric tissues.

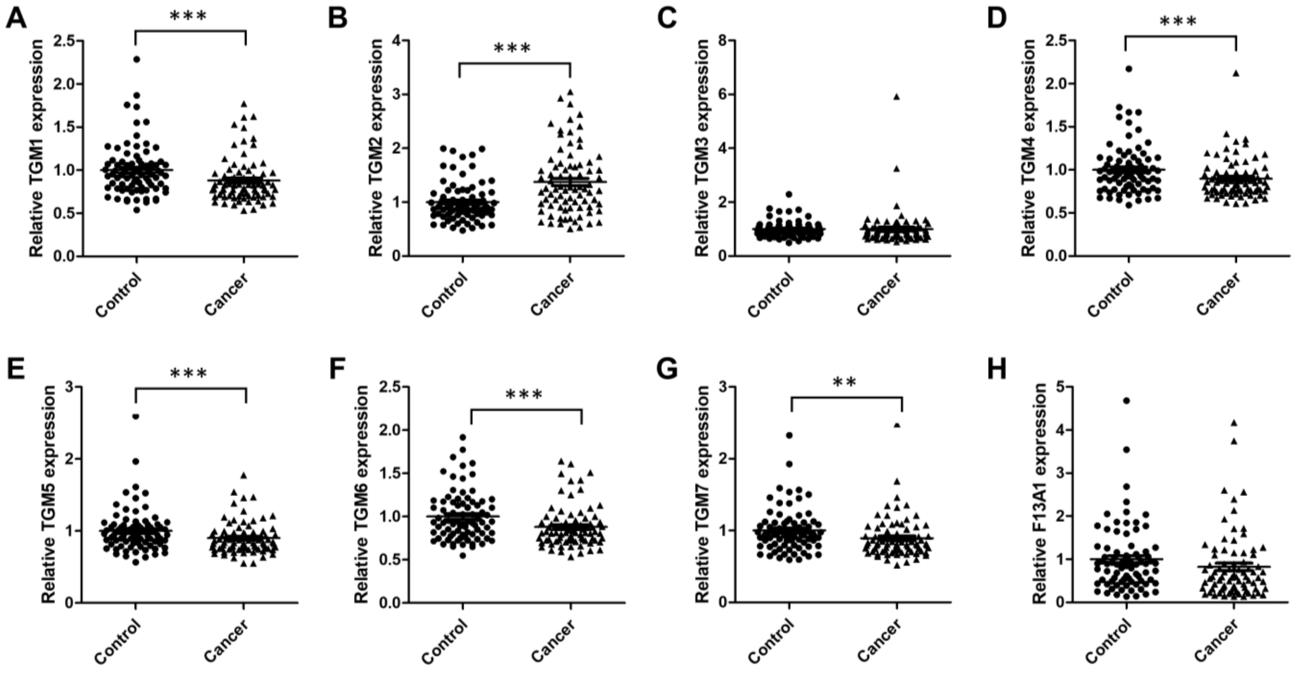

Supplementary Figure S3. The mRNA expression of transglutaminase family in normal and gastric cancer tissues. (A-H) The mRNA expression levels of TGM1 (A), TGM2 (B), TGM3 (C), TGM4 (D), TGM5 (E), TGM6 (F), TGM7 (G), and F13A1 (H) in gastric cancer tissues (cancer) and matched normal gastric tissues (control). Gene expression microarray data were downloaded from Gene Expression Omnibus (GEO) database (https://www.ncbi.nlm.nih.gov/geo; GSE27342). Relative gene expression levels were estimated compared to expression levels of normal gastric tissues, and asterisks indicate statistically significant differences (**, $P < 0.01$; ***, $P < 0.001$) compared to normal gastric tissues.

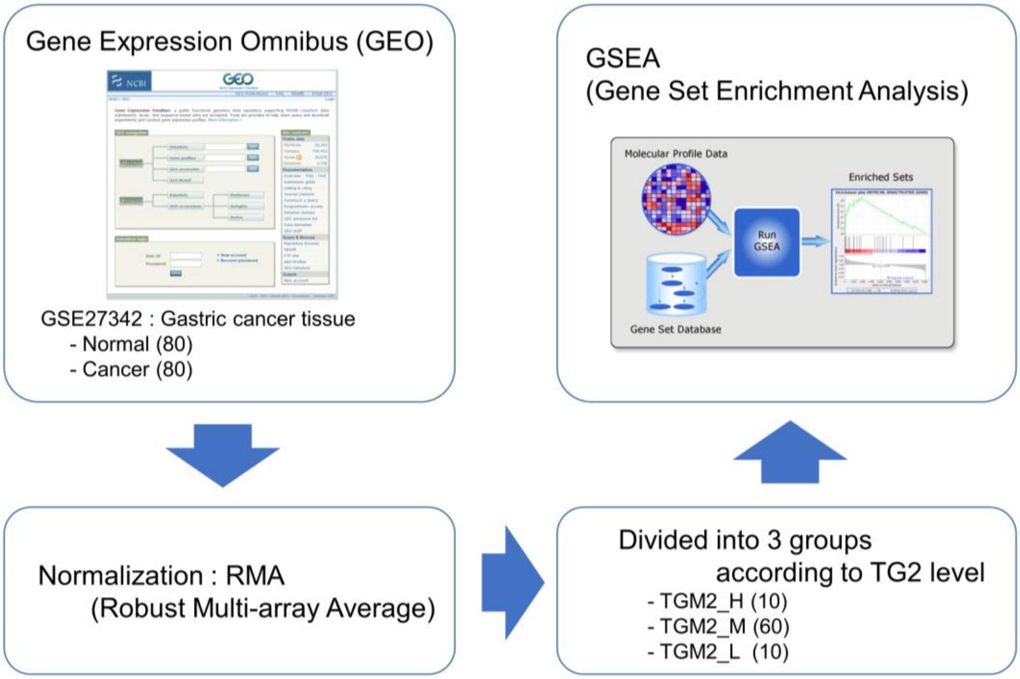

Supplementary Figure S4. The schematic summary of gene set enrichment analysis (GSEA) using gastric cancer tissue data from Expression Omnibus (GEO) database. Gene expression microarray data were downloaded from GEO database (https://www.ncbi.nlm.nih.gov/geo; GSE27342) and normalized by Robust Multi-array Average (RMA) algorithm. With selected 10 samples with highest expression levels of TGM2 (TGM2_H) and 10 samples with lowest expression levels of TGM2 (TGM2_L), GSEA between two groups was performed for gene sets from gene ontology (GO) biological process and motif gene sets for transcription factor targets.

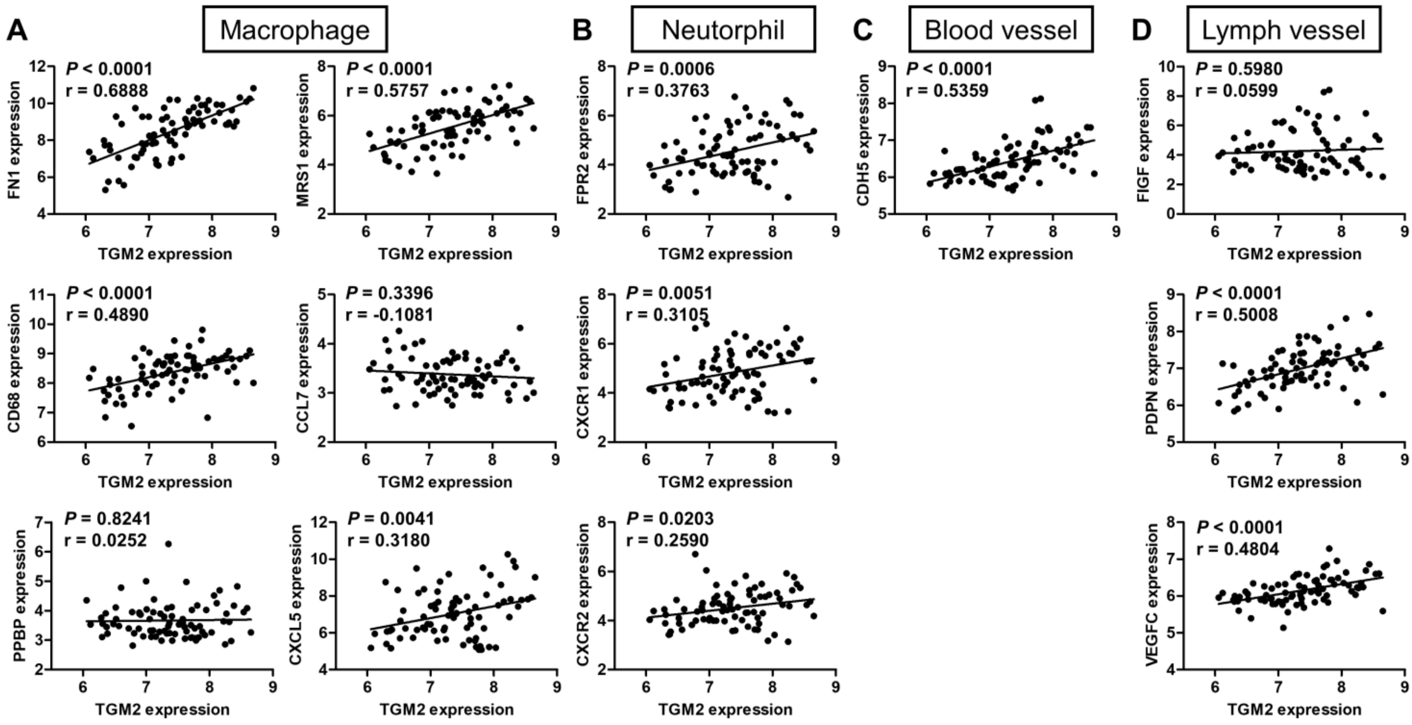

Supplementary Figure S5. Correlation of TGM2 expression with markers of macrophage, neutrophil, blood vessel and lymph vessel in 80 gastric cancer tissues. Microarray data were downloaded from Gene Expression Omnibus (GEO; GSE27342). Correlation between TGM2 expression and markers of macrophage (A), neutrophil (B), blood vessel (C) and lymph vessel (D) was estimated by linear regression analysis. P-values by linear regression and Pearson correlation coefficients (r) were indicated.

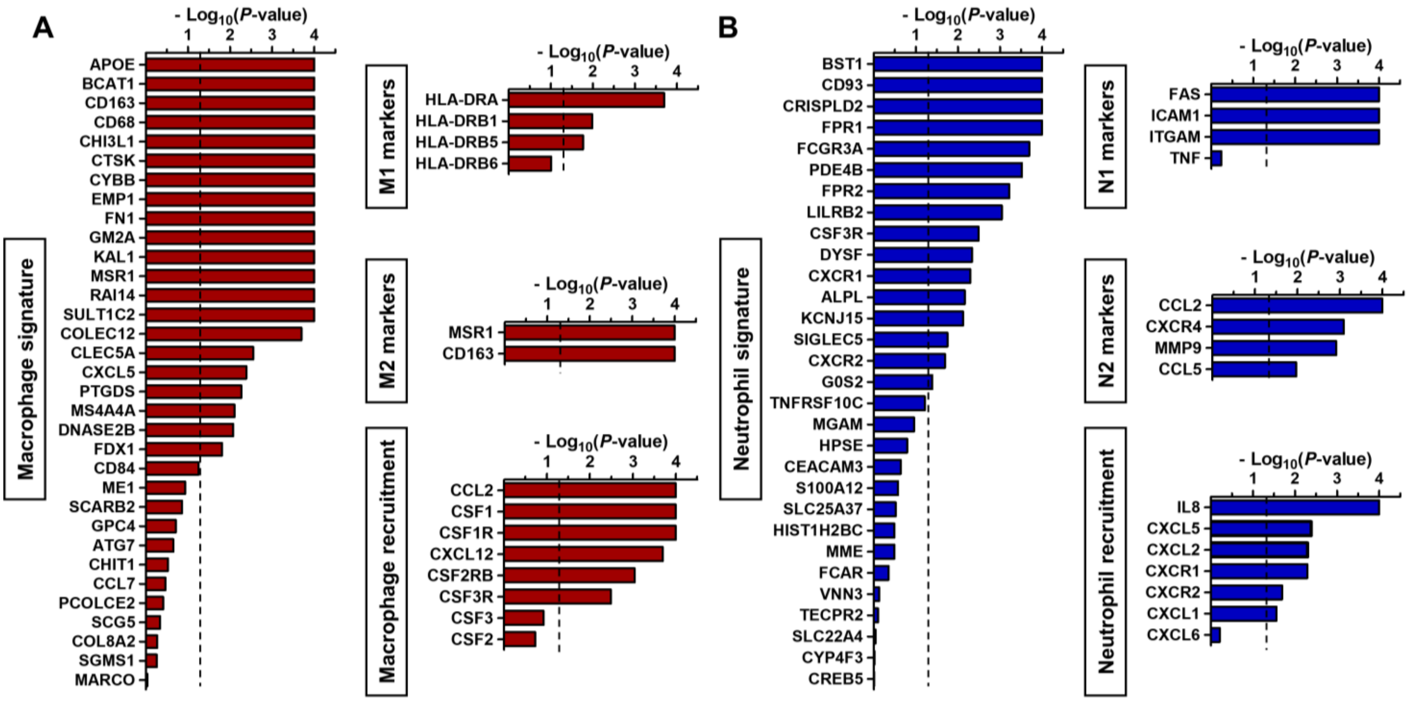

Supplementary Figure S6. Correlation of TGM2 expression with expression of genes related with macrophages (A) and neutrophils (B) in gastric cancer tissues. Gene expression microarray data were downloaded from Gene Expression Omnibus (GEO; GSE27342). Correlation between TGM2 expression and expression of genes related with macrophages and neutrophils was estimated by linear regression analysis, and the dashed line indicates a reference for statistical significance ($P < 0.05$).

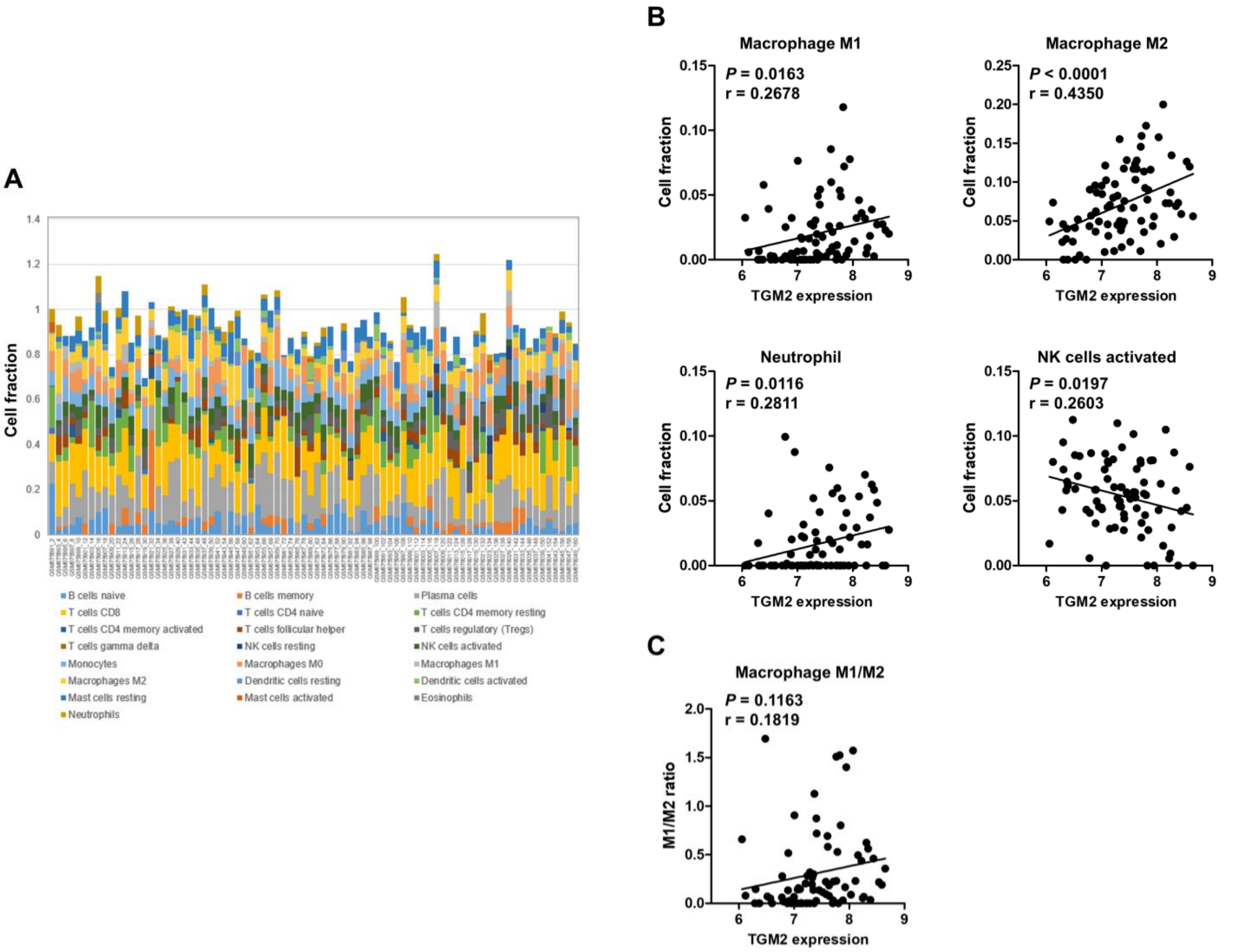

Supplementary Figure S7. Estimation of tumor-infiltrating immune cell types using CIBERSORT analysis. (A) Graphic representation of the estimation for tumor-infiltrating immune cell types analyzed using CIBERSORT. Gene expression microarray data were downloaded from Gene Expression Omnibus (GEO; GSE27342). The composition of tumor-infiltrating immune cells was calculated for each patient (x-axis) using expression microarray data from tumor tissues. (B) Correlation between TGM2 expression and abundance of M1 macrophage, M2 macrophage, neutrophil, and activated NK cells estimated using CIBERSORT. Correlation between TGM2 expression and abundance of each cell type was estimated by linear regression analysis. P-values by linear regression and Pearson correlation coefficients (r) were indicated. (C) Correlation between TGM2 expression and M1/M2 macrophage ration was estimated by linear regression analysis. P-values by linear regression and Pearson correlation coefficients (r) were indicated.

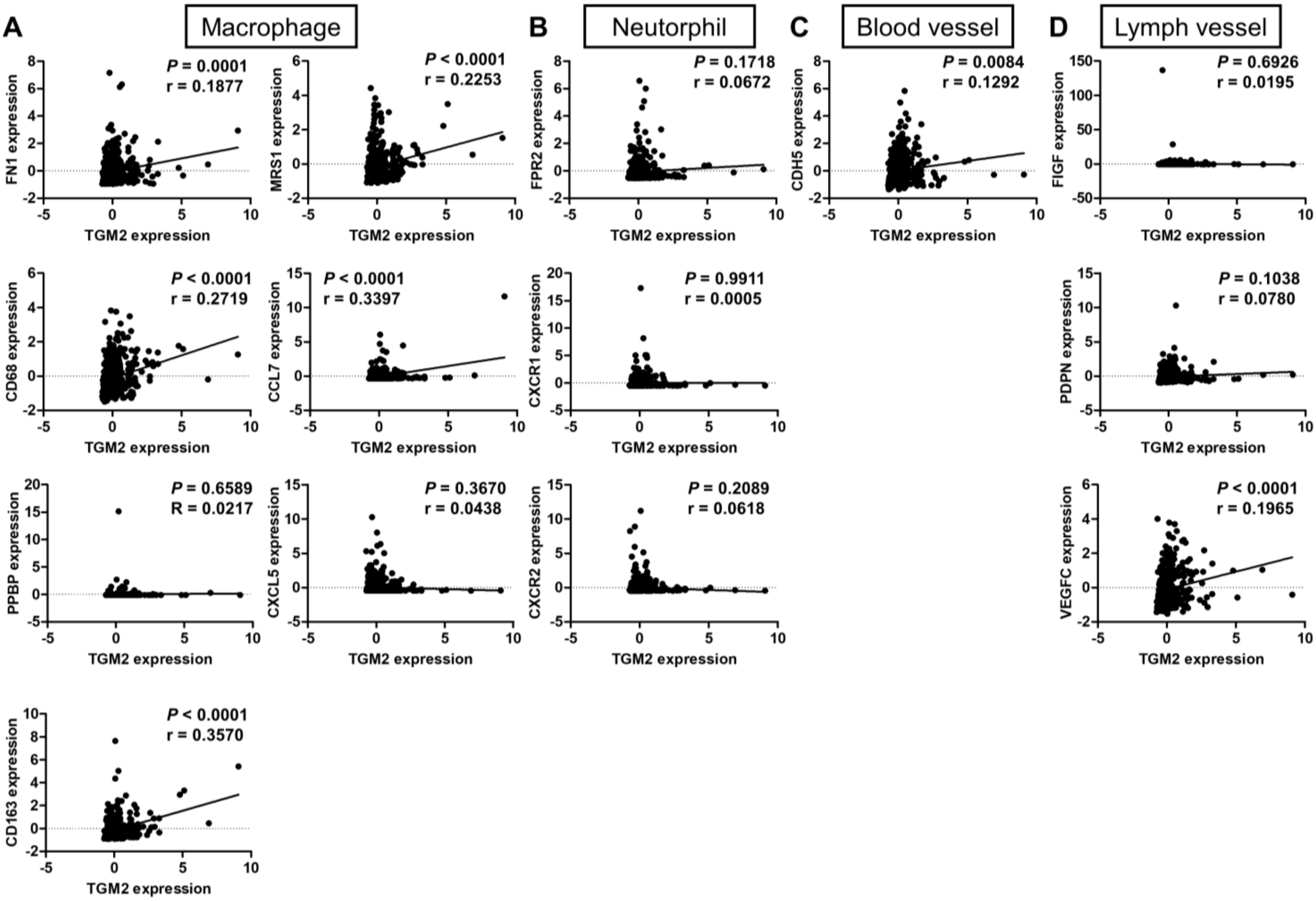

Supplementary Figure S8. Correlation of TGM2 expression with markers of macrophage, neutrophil, blood vessel and lymph vessel from TCGA database. Gene expression data from RNA sequencing were downloaded from TCGA dataset (Stomach adenocarcinoma (TCGA, Firehose Legacy, n = 478)). Correlation between TGM2 expression and markers of macrophage (A), neutrophil (B), blood vessel (C) and lymph vessel (D) was estimated by linear regression analysis. P-values by linear regression and Pearson correlation coefficients (r) were indicated.

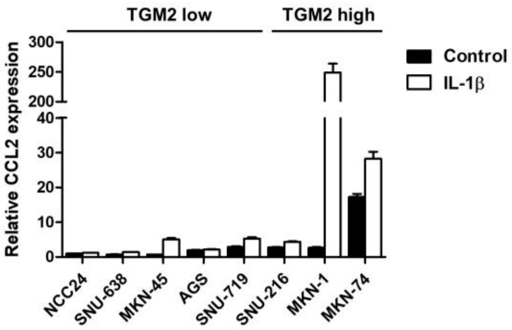

Supplementary Figure S9. Levels of mRNA for CCL2 in gastric cancer cell lines. The expressions of mRNA for CCL2 were measured by real-time PCR in control and IL-1β (10 ng/ml)-treated gastric cancer cells after 3 hr. Relative expression of CCL2 mRNA were estimated compared to untreated NCC24 cells.

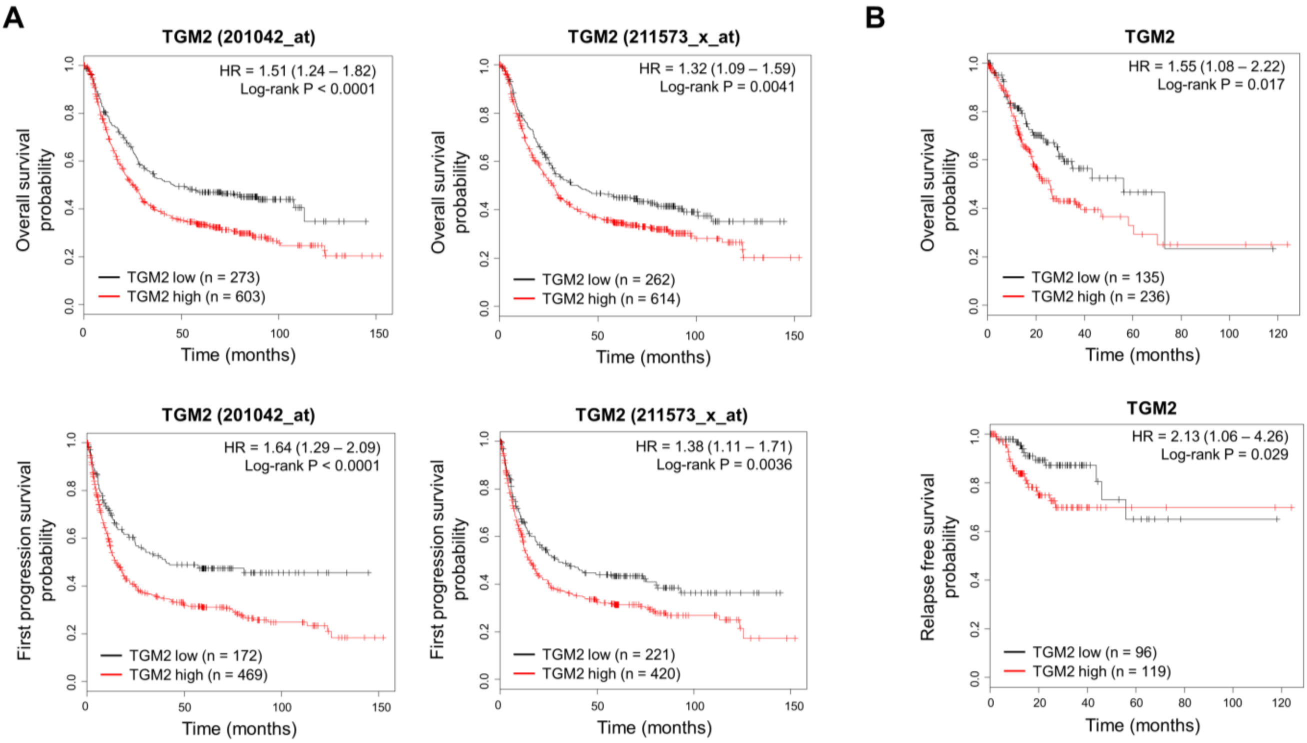

Supplementary Figure S10. Effect of TGM2 mRNA expression on the survival of GC patients. Kaplan-Meier plot were calculated for overall (upper panel) and first progression/relapse free survival (lower panel) of GC patients with high TGM2 expression (red line) and low TGM2 expression (black line) using the online bioinformatics tool Kaplan-Meier Plotter (https://kmplot.com/analysis/) (A) and TCGA database (B).

Supplementary Table S1. Copy number alterations of transglutaminase family detected by aCGH in gastric cancer samples

Gene	Chromosome No.	Location	CNA Frequency (%)	Mode of CNA
TGM1	14	24,718,320-24,732,416	0/103 (0%)	(-)
TGM2	20	36,756,864-36,793,700	16/103 (15.5%)	Amplification
TGM3	20	24,718,320-24,732,416	5/103 (4.9%)	Amplification
TGM4	3	44,916,098-44,956,088	0/103 (0%)	(-)
TGM5	15	43,524,793-43,559,055	0/103 (0%)	(-)
TGM6	20	2,361,554-2,413,399	5/103 (4.9%)	Amplification
TGM7	15	43,568,479-43,594,453	0/103 (0%)	(-)
F13A1	6	6,144,311-6,320,924	2/103 (1.9%)	Amplification

Supplementary Table S2. Clinical informations of gastric cancer samples according to CNA of TGM2

		TGM2 Amplified	TGM2 Normal
		**(n=16)**	**(n=87)**
**Age**	~50	4	9
(*P* = 0.1612)	51~60	3	21
	61~70	3	35
	71~	6	22
**Sex**	Male	12	64
(*P* = 1)	Female	4	23
**Lauren classification**	Interstinal type	11	50
(*P* = 0.6362)	Diffuse type	4	32
	Mixed type	1	4
	Unclassified	0	1
**TNM stage**	I	2	13
(*P* = 0.6293)	II	4	20
	III	9	37
	IV	1	17
**Location**	Upper stomach	5	17
(*P* = 0.4137)	Middle stomach	0	5
	Lower stomach	7	43
	Entire stomach	1	1
	Miscellaneous	3	21

P -value : Fisher's exact test

Supplementary Table S3. List of genes showing correlation with TGM2 mRNA expression

Gene Symbol	Cytoband	Pearson Score
HAPLN3	15q26.1	0.61
CX3CL1	16q21	0.57
GBP1P1	1p22.2	0.56
PDCD1LG2	9p24.1	0.54
IL18BP	11q13.4	0.53
C1QA	1p36.12	0.52
CXCL10	4q21.1	0.52
CD8A	2p11.2	0.51
GZMH	14q12	0.51
CXCL9	4q21.1	0.51
PML	15q24.1	0.51
GBP5	1p22.2	0.51
ICAM1	19p13.2	0.5
CXCL11	4q21.1	0.5
CCL5	17q12	0.49
TSPAN9	12p13.33-p13.32	0.49
C1QB	1p36.12	0.47
C1QC	1p36.12	0.47
IL2RA	10p15.1	0.47
KIFC3	16q21	0.47
NKG7	19q13.41	0.47
NPR3	5p13.3	0.47
SLA2	20q11.23	0.47
SLAMF8	1q23.2	0.47
CCR5	3p21.31	0.46
FCGR1A	1q21.2	0.46
FOXC2	16q24.1	0.46
HLA-DOA	6p21.32	0.46
CXCR6	3p21.31	0.46
TRAFD1	12q24.13	0.46
TMEM140	7q33	0.46
SAMHD1	20q11.23	0.46
TNFAIP8L3	15q21.2	0.46
FASLG	1q24.3	0.45
HLA-DPA1	6p21.32	0.45
TSPAN4	11p15.5	0.45
WARS	14q32.2	0.45
JAKMIP1	4p16.1	0.45
LAMP3	3q27.1	0.45
ZNF683	1p36.11	0.45
VGLL3	3p12.1	0.45
CD14	5q31.3	0.44
TYMP	22q13.33	0.44
NINJ1	9q22.31	0.44
MAFB	20q12	0.44
LAP3	4p15.32	0.44
TMTC1	12p11.22	0.44
TBX21	17q21.32	0.44
SERPING1	11q12.1	0.43

CTSW	11q13.1	0.43
GBP1	1p22.2	0.43
LAG3	12p13.31	0.43
PRF1	10q22.1	0.43
CEBPB	20q13.13	0.42
FCGR3A	1q23.3	0.42
IL15RA	10p15.1	0.42
DOK2	8p21.3	0.42
APOL3	22q12.3	0.42
BATF2	11q13.1	0.42
NFAM1	22q13.2	0.42
IFNL1	19q13.2	0.42
CHRNA9	4p14	0.42
MKL1	22q13.1-q13.2	0.42
APOBEC3G	22q13.1	0.42
BICD1	12p11.21	0.41
GBP2	1p22.2	0.41
GGT5	22q11.23	0.41
GZMB	14q12	0.41
IL2RB	22q12.3	0.41
MSN	Xq12	0.41
TEAD4	12p13.33	0.41
TMEM86A	11p15.1	0.41
HRCT1	9p13.3	0.41
GPR139	16p12.3	0.41
GJD3	17q21.2	0.41
APOBEC3H	22q13.1	0.4
CD4	12p13.31	0.4
CD40	20q13.12	0.4
IFNG	12q15	0.4
LAIR1	19q13.42	0.4
SERPINE1	7q22.1	0.4
SELPLG	12q24.11	0.4
STX11	6q24.2	0.4
PLEKHO2	15q22.31	0.4
CALHM6	6q22.1	0.4
WNT5B	12p13.33	0.4
ZBP1	20q13.31	0.4
C1RL	12p13.31	0.4
BATF3	1q32.3	0.4
HEG1	3q21.2	0.4
PLA2G2D	1p36.12	0.4
CD209	19p13.2	0.4
CD38	4p15.32	0.39
IL12RB1	19p13.11	0.39
RARRES3	11q12.3	0.39
LGALS17A	19q13.2	0.39
UBD	6p22.1	0.39
FCRL6	1q23.2	0.39
GIMAP5	7q36.1	0.39
ATP10A	15q12	0.39
AIF1	6p21.33	0.38

AOAH	7p14.2	0.38
CD74	5q33.1	0.38
FCGR1B	1p11.2	0.38
FCGR2A	1q23.3	0.38
GALNT2	1q42.13	0.38
CXCR3	Xq13.1	0.38
HLA-DPB1	6p21.32	0.38
CXCR2P1	2q35	0.38
IRF1	5q31.1	0.38
NNMT	11q23.2	0.38
PAPPA	9q33.1	0.38
ST3GAL2	16q22.1	0.38
UBE2L6	11q12.1	0.38
TRIM22	11p15.4	0.38
LILRB4	19q13.42	0.38
CD244	1q23.3	0.38
CRTAM	11q24.1	0.38
PLA2G15	16q22.1	0.38
SLAMF7	1q23.3	0.38
FCGR1CP	1q21.1	0.38
GPR171	3q25.1	0.38
DCP1B	12p13.33	0.38
CD2	1p13.1	0.37
CMKLR1	12q23.3	0.37
HLA-E	6p22.1	0.37
IFNAR2	21q22.11	0.37
ITGAE	17p13.2	0.37
TAP2	6p21.32	0.37
TCN2	22q12.2	0.37
PPP1R18	6p21.33	0.37
R3HDML	20q13.12	0.37
TNFSF13B	13q33.3	0.37
IL27	16p12.1-p11.2	0.37
SLC15A3	11q12.2	0.37
HAVCR2	5q33.3	0.37
ITGA11	15q23	0.37
PLD3	19q13.2	0.37
DENND5B	12p11.21	0.37
AXL	19q13.2	0.36
CCR1	3p21.31	0.36
CCR8	3p22.1	0.36
CSF1	1p13.3	0.36
PHC2	1p35.1	0.36
HLA-DRA	6p21.32	0.36
TNFRSF9	1p36.23	0.36
CIITA	16p13.13	0.36
MYO1F	19p13.2	0.36
TIFAB	5q31.1	0.36
SNAI2	8q11.21	0.36
SIGLEC1	20p13	0.36
STAT1	2q32.2	0.36
STAT2	12q13.3	0.36

XCL2	1q24.2	0.36
TGFBI	5q31.1	0.36
TNFRSF4	1p36.33	0.36
CD163	12p13.31	0.36
LILRB2	19q13.42	0.36
VAMP5	2p11.2	0.36
HCST	19q13.12	0.36
AZIN2	1p35.1	0.36
TLR8	Xp22.2	0.36
ADA2	22q11.1	0.36
SLC9A7	Xp11.3|Xp11.3	0.36
GIMAP4	7q36.1	0.36
DRAM1	12q23.2	0.36
ERC1	12p13.33	0.36
STAB1	3p21.1	0.36
ZNF319	16q21	0.36
PREX1	20q13.13	0.36
C1S	12p13.31	0.35
C3	19p13.3	0.35
CD3G	11q23.3	0.35
IFI35	17q21.31	0.35
IL9R	Xq28 and Yq12	0.35
IDO1	8p11.21	0.35
TAP1	6p21.32	0.35
TNFRSF1B	1p36.22	0.35
THEMIS2	1p35.3	0.35
RHNO1	12p13.33	0.35
ERGIC2	12p11.22	0.35
P4HA3	11q13.4	0.35
KLRK1	12p13.2	0.35
ADAP2	17q11.2	0.35
APOBEC3C	22q13.1	0.35
ALX4	11p11.2	0.35
IL4I1	19q13.33	0.35
C1R	12p13.31	0.34
CSF1R	5q32	0.34
FYN	6q21	0.34
GSTT2	22q11.23	0.34
HLA-DRB1	6p21.32	0.34
IFIT3	10q23.31	0.34
JAK2	9p24.1	0.34
KLRD1	12p13.2	0.34
LIMK1	7q11.23	0.34
MEFV	16p13.3	0.34
CCL7	17q12	0.34
PSTPIP1	15q24.3	0.34
CD96	3q13.13-q13.2	0.34
IFI30	19p13.11	0.34
LILRB1	19q13.42	0.34
RASSF8	12p12.1	0.34
VSIG4	Xq12	0.34
IGFLR1	19q13.12	0.34

SPSB1	1p36.22	0.34
APOL6	22q12.3	0.34
GBP4	1p22.2	0.34
FOXP3	Xp11.23	0.34
GIMAP8	7q36.1	0.34
SFMBT2	10p14	0.34
CMTM3	16q22.1	0.34
CACNG7	19q13.42	0.34
LRRC25	19p13.11	0.34
SYTL3	6q25.3	0.34
CD274	9p24.1	0.34
APCDD1L	20q13.32	0.33
SLC31A2	9q32	0.33
GNGT2	17q21.32	0.33
GPR25	1q32.1	0.33
GZMA	5q11.2	0.33
HCK	20q11.21	0.33
ITGB2	21q22.3	0.33
SNTB2	16q22.1	0.33
SPN	16p11.2	0.33
TULP3	12p13.33	0.33
CCR2	3p21.31	0.33
KYNU	2q22.2	0.33
LITAF	16p13.13	0.33
HPS5	11p15.1	0.33
TNFAIP8L2	1q21.3	0.33
GIMAP6	7q36.1	0.33
FAM163A	1q25.2	0.33
ETV7	6p21.31	0.33
HDAC7	12q13.11	0.33
UBASH3A	21q22.3	0.33
SIRPG	20p13	0.33
GPR141	7p14.1	0.33
MMP25	16p13.3	0.33
CD3D	11q23.3	0.32
COL8A1	3q12.1	0.32
FCER1G	1q23.3	0.32
FPR3	19q13.41	0.32
GNAI2	3p21.31	0.32
GZMM	19p13.3	0.32
HLA-C	6p21.33	0.32
HLA-DMB	6p21.32	0.32
ITPR2	12p11.23	0.32
LCP2	5q35.1	0.32
TIGIT	3q13.31	0.32
P2RY6	11q13.4	0.32
HTRA1	10q26.13	0.32
CCL4	17q12	0.32
TFE3	Xp11.23	0.32
TYROBP	19q13.12	0.32
OASL	12q24.31	0.32
TNFSF14	19p13.3	0.32

NRP1	10p11.22	0.32
CFLAR	2q33.1	0.32
GRAP2	22q13.1	0.32
APOBEC3D	22q13.1	0.32
SH2B3	12q24.12	0.32
GLIPR1	12q21.2	0.32
IL21R	16p12.1	0.32
P2RY13	3q25.1	0.32
PARP14	3q21.1	0.32
PI4K2A	10q24.2	0.32
CLDN14	21q22.13	0.32
GRAMD1A	19q13.11	0.32
THEMIS	6q22.33	0.32
MS4A6A	11q12.2	0.32
WNK1	12p13.33	0.32
ACP2	11p11.2|11p12-p11	0.31
CD3E	11q23.3	0.31
CD6	11q12.2	0.31
CD86	3q13.33	0.31
TPP1	11p15.4	0.31
HLA-DQA1	6p21.32	0.31
P2RX7	12q24.31	0.31
SCN1B	19q13.11	0.31
SMPD1	11p15.4	0.31
SOD2	6q25.3	0.31
SPI1	11p11.2	0.31
GIMAP1	7q36.1	0.31
TAPBP	6p21.32	0.31
TGFB1	19q13.2	0.31
TNFRSF1A	12p13.31	0.31
VCAM1	1p21.2	0.31
SOCS1	16p13.13	0.31
WISP1	8q24.22	0.31
IGSF6	16p12.2	0.31
IFITM3	11p15.5	0.31
BATF	14q24.3	0.31
TMEM173	5q31.2	0.31
IRX3	16q12.2	0.31
KIRREL3	11q24.2	0.31
ITPRIP	10q25.1	0.31
WWC3	Xp22.2	0.31
CYSLTR2	13q14.2	0.31
ZNFX1	20q13.13	0.31
ACP5	19p13.2	0.3
CD247	1q24.2	0.3
CYBB	Xp21.1-p11.4	0.3
GAA	17q25.3	0.3
HK3	5q35.2	0.3
HLA-DMA	6p21.32	0.3
HMOX1	22q12.3	0.3
IFI16	1q23.1	0.3
MGAT1	5q35.3	0.3

OAS2	12q24.13	0.3
STAT3	17q21.2	0.3
EOMES	3p24.1	0.3
PTGES	9q34.11	0.3
SUSD6	14q24.1	0.3
OPTN	10p13	0.3
NOD1	7p14.3	0.3
SLCO2B1	11q13.4	0.3
USB1	16q21	0.3
CD300LF	17q25.1	0.3
TOX2	20q13.12	0.3
SYDE1	19p13.12	0.3
HELZ2	20q13.33	0.3
EWSAT1	15q23	0.3
JADE2	5q31.1	0.3
JPH3	16q24.2	0.3
PXDC1	6p25.2	0.3
UBE2Z	17q21.32	0.3

Supplementary Table S4. Enriched gene ontologies in genes showing correlation with TGM2 mRNA expression

GO term	Gene count	*P* -Value	Fold Enrichment	FDR
immune response	60	2.80E-36	8.1	4.70E-33
inflammatory response	49	1.80E-27	7.3	3.10E-24
interferon-gamma-mediated signaling pathway	24	2.30E-23	19.1	4.00E-20
defense response to virus	28	1.00E-18	9.6	1.70E-15
innate immune response	40	2.80E-17	5.3	4.80E-14
regulation of immune response	27	8.20E-17	8.6	1.90E-13
adaptive immune response	25	1.10E-16	9.6	1.90E-13
T cell costimulation	19	1.40E-15	13.8	2.30E-12
positive regulation of T cell proliferation	16	9.00E-14	15.1	1.50E-10
chemokine-mediated signaling pathway	15	2.00E-11	11.9	3.40E-08
negative regulation of T cell proliferation	12	2.40E-11	18.3	4.10E-08
cell surface receptor signaling pathway	25	1.10E-10	5.2	1.90E-07
type I interferon signaling pathway	13	1.00E-09	11.5	1.80E-06
cellular response to lipopolysaccharide	16	1.30E-09	8	2.30E-06
antigen processing and presentation	12	2.50E-09	12.3	4.30E-06
signal transduction	51	3.30E-09	2.5	5.60E-06
chemotaxis	16	3.90E-09	7.4	6.70E-06
response to lipopolysaccharide	18	5.00E-09	6.2	8.60E-06
T cell receptor signaling pathway	17	7.80E-09	6.5	1.30E-05
antigen processing and presentation of peptide or polysaccharide antigen via MHC class II	8	8.20E-09	26.6	1.40E-05
JAK-STAT cascade	9	6.20E-08	15.9	1.00E-04
response to virus	14	7.00E-08	7.2	1.20E-04
negative regulation of interferon-gamma production	8	4.20E-07	16.2	7.20E-04
cellular response to interferon-gamma	10	6.20E-07	9.9	1.10E-03
myeloid dendritic cell differentiation	7	6.40E-07	20.8	1.10E-03
cell adhesion	26	6.40E-07	3.2	1.10E-03
positive regulation of interferon-gamma production	9	1.20E-06	11.1	2.10E-03
T cell activation	9	1.50E-06	10.8	2.50E-03
leukocyte migration	13	1.70E-06	6	2.80E-03
positive regulation of ERK1 and ERK2 cascade	15	2.80E-06	4.8	4.70E-03
cell-cell signaling	18	2.80E-06	4	4.80E-03
antigen processing and presentation of exogenous peptide antigen via MHC class II	11	5.00E-06	6.8	8.50E-03
positive regulation of monocyte chemotaxis	6	6.10E-06	21.2	1.00E-02
neutrophil chemotaxis	9	2.00E-05	7.7	3.40E-02
cytokine-mediated signaling pathway	12	2.10E-05	5.2	3.50E-02
cytolysis	6	2.70E-05	16.2	4.50E-02
apoptotic signaling pathway	9	3.40E-05	7.2	5.80E-02
positive regulation of inflammatory response	9	4.20E-05	7	7.20E-02
peptide antigen assembly with MHC class II protein complex	4	5.30E-05	45.2	9.00E-02
response to interferon-gamma	6	5.30E-05	14.1	9.00E-02
leukocyte cell-cell adhesion	6	6.50E-05	13.6	1.10E-01
cellular response to organic cyclic compound	8	7.70E-05	7.7	1.30E-01
cellular response to ethanol	5	8.20E-05	20.2	1.40E-01
extrinsic apoptotic signaling pathway	7	8.80E-05	9.4	1.50E-01
positive regulation of nitric oxide biosynthetic process	7	1.00E-04	9.2	1.70E-01
positive regulation of cAMP metabolic process	4	1.00E-04	37.7	1.80E-01
positive regulation of tumor necrosis factor production	7	1.70E-04	8.4	2.80E-01
apoptotic process	24	1.90E-04	2.4	3.20E-01
tumor necrosis factor-mediated signaling pathway	10	2.40E-04	4.8	4.10E-01
negative regulation of transposition	4	2.80E-04	28.3	4.80E-01
transmembrane receptor protein tyrosine kinase signaling pathway	9	2.90E-04	5.3	5.00E-01
positive regulation of interleukin-4 production	5	3.70E-04	14.1	6.20E-01
natural killer cell activation	5	3.70E-04	14.1	6.20E-01
positive regulation of T cell apoptotic process	4	4.20E-04	25.1	7.10E-01
positive regulation of defense response to virus by host	5	5.40E-04	12.8	9.10E-01
positive regulation of I-kappaB kinase/NF-kappaB signaling	11	5.80E-04	3.9	9.80E-01
negative regulation of viral genome replication	6	6.50E-04	8.5	1.10E+00

cellular defense response	7	7.70E-04	6.4	1.30E+00
negative regulation of angiogenesis	7	7.70E-04	6.4	1.30E+00
negative regulation of interleukin-2 production	4	8.10E-04	20.6	1.40E+00
protein complex assembly	9	1.00E-03	4.4	1.80E+00
negative regulation of interleukin-10 production	4	1.10E-03	18.8	1.80E+00
negative regulation of interleukin-12 production	4	1.10E-03	18.8	1.80E+00
positive regulation of cAMP-mediated signaling	4	1.10E-03	18.8	1.80E+00
regulation of interferon-gamma-mediated signaling pathway	4	1.40E-03	17.4	2.30E+00
regulation of apoptotic process	12	1.40E-03	3.2	2.40E+00
cellular response to interleukin-1	7	1.60E-03	5.6	2.60E+00
positive regulation of cell migration	11	1.60E-03	3.4	2.70E+00
antigen processing and presentation of peptide antigen via MHC class I	5	1.80E-03	9.4	3.00E+00
heterophilic cell-cell adhesion via plasma membrane cell adhesion molecules	6	1.80E-03	6.8	3.10E+00
complement activation, classical pathway	8	1.80E-03	4.6	3.10E+00
lipopolysaccharide-mediated signaling pathway	5	2.30E-03	8.8	3.80E+00
negative regulation of extrinsic apoptotic signaling pathway via death domain receptors	5	2.60E-03	8.6	4.30E+00
positive regulation of cytosolic calcium ion concentration	9	2.60E-03	3.8	4.30E+00
negative regulation of inflammatory response	7	2.70E-03	5	4.50E+00
viral entry into host cell	7	2.90E-03	4.9	4.80E+00
membrane to membrane docking	3	3.00E-03	33.9	5.00E+00
dendritic cell chemotaxis	4	3.10E-03	13.3	5.10E+00
positive regulation of peptidyl-tyrosine phosphorylation	7	3.30E-03	4.8	5.40E+00
cellular response to tumor necrosis factor	8	3.30E-03	4.1	5.50E+00
positive regulation of T cell activation	4	3.60E-03	12.6	6.00E+00
hemopoiesis	6	3.80E-03	5.7	6.20E+00
movement of cell or subcellular component	7	4.10E-03	4.6	6.80E+00
defense response to protozoan	4	4.30E-03	11.9	7.00E+00
positive regulation of angiogenesis	8	4.30E-03	3.9	7.00E+00
positive regulation of MHC class I biosynthetic process	3	4.40E-03	28.3	7.30E+00
positive regulation of B cell proliferation	5	4.80E-03	7.2	7.80E+00
positive regulation of calcium-mediated signaling	4	4.90E-03	11.3	8.10E+00
cell chemotaxis	6	5.70E-03	5.2	9.30E+00
antigen processing and presentation of endogenous peptide antigen via MHC class I	3	6.10E-03	24.2	9.90E+00
positive regulation of natural killer cell chemotaxis	3	6.10E-03	24.2	9.90E+00
negative regulation of single stranded viral RNA replication via double stranded DNA intermediate	3	6.10E-03	24.2	9.90E+00
DNA cytosine deamination	3	6.10E-03	24.2	9.90E+00
monocyte chemotaxis	5	6.30E-03	6.7	1.00E+01
Fc-gamma receptor signaling pathway involved in phagocytosis	8	7.30E-03	3.6	1.20E+01
response to drug	13	7.90E-03	2.4	1.30E+01
positive regulation of isotype switching to IgG isotypes	3	8.10E-03	21.2	1.30E+01
T cell chemotaxis	3	8.10E-03	21.2	1.30E+01
positive regulation of interleukin-1 beta production	3	8.10E-03	21.2	1.30E+01
cellular response to mechanical stimulus	6	8.30E-03	4.8	1.30E+01
response to electrical stimulus	4	8.30E-03	9.4	1.30E+01
aging	9	9.00E-03	3.1	1.40E+01
positive regulation of NF-kappaB transcription factor activity	8	9.30E-03	3.4	1.50E+01
intrinsic apoptotic signaling pathway in response to DNA damage	5	9.30E-03	6	1.50E+01
response to interferon-beta	3	1.00E-02	18.8	1.60E+01
negative regulation of activated T cell proliferation	3	1.00E-02	18.8	1.60E+01
cytidine deamination	3	1.00E-02	18.8	1.60E+01
negative regulation of cell proliferation	15	1.10E-02	2.1	1.70E+01
negative regulation of gene expression	8	1.10E-02	3.3	1.70E+01
calcium ion transport	6	1.10E-02	4.5	1.70E+01
T cell homeostasis	4	1.20E-02	8.4	1.80E+01
positive regulation of release of sequestered calcium ion into cytosol	4	1.20E-02	8.4	1.80E+01

regulation of cell shape	8	1.20E-02	3.2	1.90E+01
positive regulation of type I interferon production	5	1.20E-02	5.5	1.90E+01
positive regulation of T cell migration	3	1.30E-02	17	2.00E+01
response to interferon-alpha	3	1.30E-02	17	2.00E+01
positive regulation of T cell chemotaxis	3	1.30E-02	17	2.00E+01
regulation of defense response to virus by virus	4	1.30E-02	8.1	2.00E+01
negative regulation of DNA binding	4	1.30E-02	8.1	2.00E+01
response to cytokine	5	1.30E-02	5.4	2.00E+01
negative regulation of smooth muscle cell proliferation	4	1.40E-02	7.8	2.10E+01
negative regulation of T cell activation	3	1.50E-02	15.4	2.30E+01
immunoglobulin mediated immune response	3	1.50E-02	15.4	2.30E+01
positive regulation of macrophage chemotaxis	3	1.50E-02	15.4	2.30E+01
positive regulation of tumor necrosis factor biosynthetic process	3	1.50E-02	15.4	2.30E+01
positive regulation of tyrosine phosphorylation of Stat1 protein	3	1.50E-02	15.4	2.30E+01
negative thymic T cell selection	3	1.50E-02	15.4	2.30E+01
negative regulation of interleukin-17 production	3	1.50E-02	15.4	2.30E+01
T cell differentiation	4	1.50E-02	7.5	2.30E+01
response to gamma radiation	4	1.70E-02	7.3	2.50E+01
cellular response to ionizing radiation	4	1.70E-02	7.3	2.50E+01
regulation of cell proliferation	9	1.70E-02	2.8	2.50E+01
defense response to Gram-positive bacterium	6	1.70E-02	4	2.50E+01
viral process	12	1.70E-02	2.3	2.60E+01
dendritic cell differentiation	3	1.80E-02	14.1	2.70E+01
positive regulation of interleukin-2 biosynthetic process	3	1.80E-02	14.1	2.70E+01
negative regulation of immune response	3	1.80E-02	14.1	2.70E+01
mesoderm development	4	1.80E-02	7.1	2.70E+01
complement activation	6	1.90E-02	3.9	2.80E+01
protein kinase B signaling	4	2.00E-02	6.9	2.90E+01
negative regulation of calcium ion transport	3	2.10E-02	13	3.10E+01
positive regulation of T cell mediated cytotoxicity	3	2.10E-02	13	3.10E+01
regulation of protein phosphorylation	4	2.20E-02	6.7	3.10E+01
cellular calcium ion homeostasis	6	2.40E-02	3.6	3.40E+01
G-protein coupled purinergic nucleotide receptor signaling pathway	3	2.50E-02	12.1	3.40E+01
positive regulation of transcription from RNA polymerase II promoter	27	2.50E-02	1.6	3.50E+01
antigen processing and presentation of exogenous peptide antigen via MHC class I, TAP-dependent	5	2.50E-02	4.5	3.50E+01
intracellular signal transduction	14	2.70E-02	2	3.70E+01
intrinsic apoptotic signaling pathway in response to oxidative stress	3	2.80E-02	11.3	3.80E+01
G-protein coupled receptor signaling pathway	25	2.80E-02	1.6	3.90E+01
negative regulation of tumor necrosis factor production	4	2.90E-02	6	3.90E+01
phospholipase C-activating G-protein coupled receptor signaling pathway	5	2.90E-02	4.3	3.90E+01
negative regulation of neuron apoptotic process	7	3.00E-02	3	4.00E+01
defense response	5	3.00E-02	4.2	4.10E+01
integrin-mediated signaling pathway	6	3.10E-02	3.4	4.10E+01
cell death	4	3.10E-02	5.8	4.10E+01
cellular response to interferon-beta	3	3.20E-02	10.6	4.20E+01
proteolysis	16	3.20E-02	1.8	4.30E+01
negative regulation of myoblast fusion	2	3.50E-02	56.5	4.50E+01
CD8-positive, alpha-beta T cell differentiation involved in immune response	2	3.50E-02	56.5	4.50E+01
negative regulation of interleukin-12 secretion	2	3.50E-02	56.5	4.50E+01
cytosol to ER transport	2	3.50E-02	56.5	4.50E+01
leukocyte adhesive activation	2	3.50E-02	56.5	4.50E+01
positive regulation of T cell anergy	2	3.50E-02	56.5	4.50E+01
polysaccharide assembly with MHC class II protein complex	2	3.50E-02	56.5	4.50E+01
antigen processing and presentation of exogenous peptide antigen via MHC class I	2	3.50E-02	56.5	4.50E+01
positive regulation of growth factor dependent skeletal muscle satellite cell proliferation	2	3.50E-02	56.5	4.50E+01

positive regulation of type IIa hypersensitivity	2	3.50E-02	56.5	4.50E+01
vascular endothelial growth factor receptor signaling pathway	5	3.80E-02	3.9	4.80E+01
positive regulation of histone acetylation	3	3.90E-02	9.4	4.90E+01
response to vitamin D	3	3.90E-02	9.4	4.90E+01
cellular response to extracellular stimulus	3	3.90E-02	9.4	4.90E+01
positive regulation of leukocyte chemotaxis	3	3.90E-02	9.4	4.90E+01
macrophage differentiation	3	3.90E-02	9.4	4.90E+01
positive regulation of natural killer cell mediated cytotoxicity	3	3.90E-02	9.4	4.90E+01
positive regulation of innate immune response	3	3.90E-02	9.4	4.90E+01
response to nutrient	5	4.20E-02	3.8	5.10E+01
positive regulation of gene expression	10	4.30E-02	2.2	5.30E+01
T cell proliferation	3	4.30E-02	8.9	5.30E+01
positive regulation of osteoclast differentiation	3	4.30E-02	8.9	5.30E+01
defense response to bacterium	7	4.40E-02	2.7	5.30E+01
positive regulation of interleukin-6 production	4	4.50E-02	5	5.40E+01
blood circulation	4	4.50E-02	5	5.40E+01
positive regulation of pri-miRNA transcription from RNA polymerase II promoter	3	4.80E-02	8.5	5.60E+01
superoxide metabolic process	3	4.80E-02	8.5	5.60E+01
positive regulation of smooth muscle cell migration	3	4.80E-02	8.5	5.60E+01
positive regulation of fat cell differentiation	4	5.00E-02	4.8	5.80E+01
